# Supplementary material for: Detection and spread of high pathogenicity avian influenza virus H5N1 in the Antarctic Region
Source: Nat Commun. 2024 Sep 3;15:7433. doi: 10.1038/s41467-024-51490-8 (PMC11372179; doi:10.1038/s41467-024-51490-8)
Supplement: Supplementary file 1 — Supplementary Information [file 41467_2024_51490_MOESM1_ESM.pdf]

Supplementary Figure 1. Phylogenetic analysis of all influenza A virus gene segments from detections in the Falkland Islands and South Georgia. Representative global H5N1 HPAIV sequences collected since 1st September 2020 were combined with the sequences generated from samples collected from South Georgia and the Falkland Islands and used to infer maximum likelihood phylogenetic trees of (**A**) PB2, PB1, PA and HA and (**B**) NP, NA, MP and NS. Tips are coloured according to region of origin, and the sequences from South Georgia and the Falkland Islands are indicated.

A

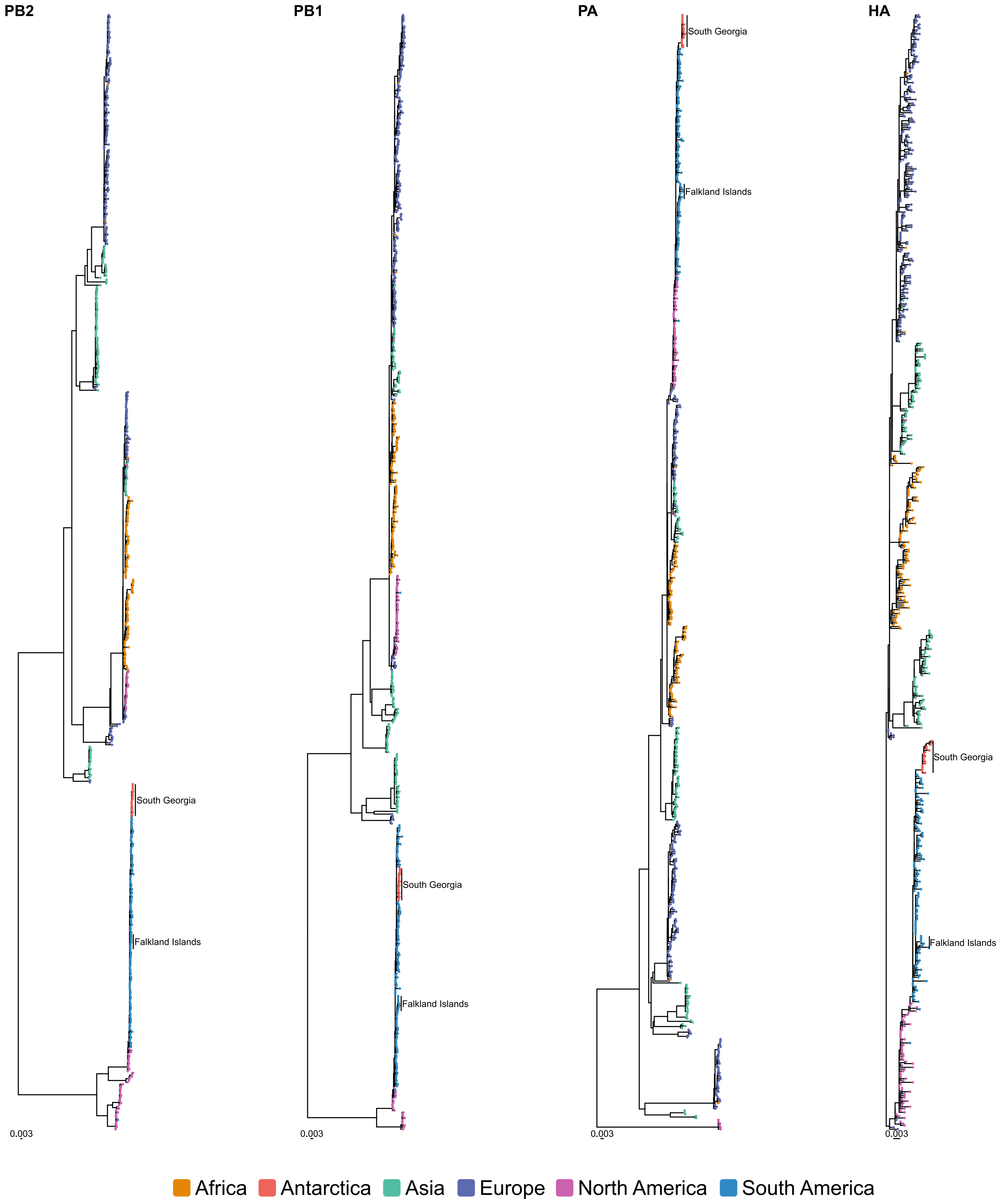

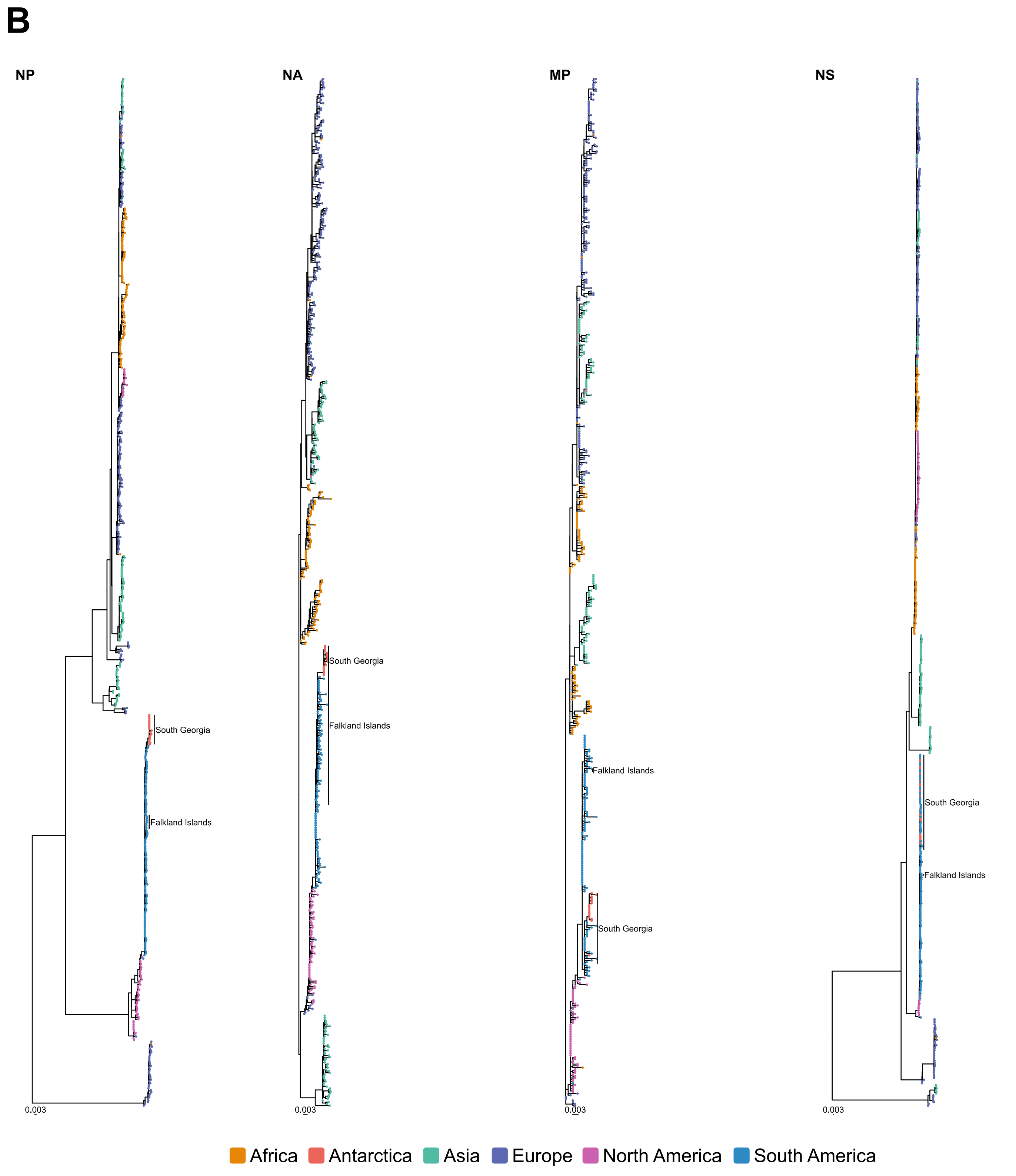

B
NP
NA
MP
NS
South Georgia
Falkland Islands
South Georgia
Falkland Islands
Falkland Islands
South Georgia
South Georgia
Falkland Islands
0.003
0.003
0.003
0.003
Africa
Antarctica
Asia
Europe
North America
South America

Supplementary Figure 2. Extended maximum clade credibility (MCC) phylogeny of the HA gene by BEAST analysis. Extension to Figure 2A including strain names at branch termini.

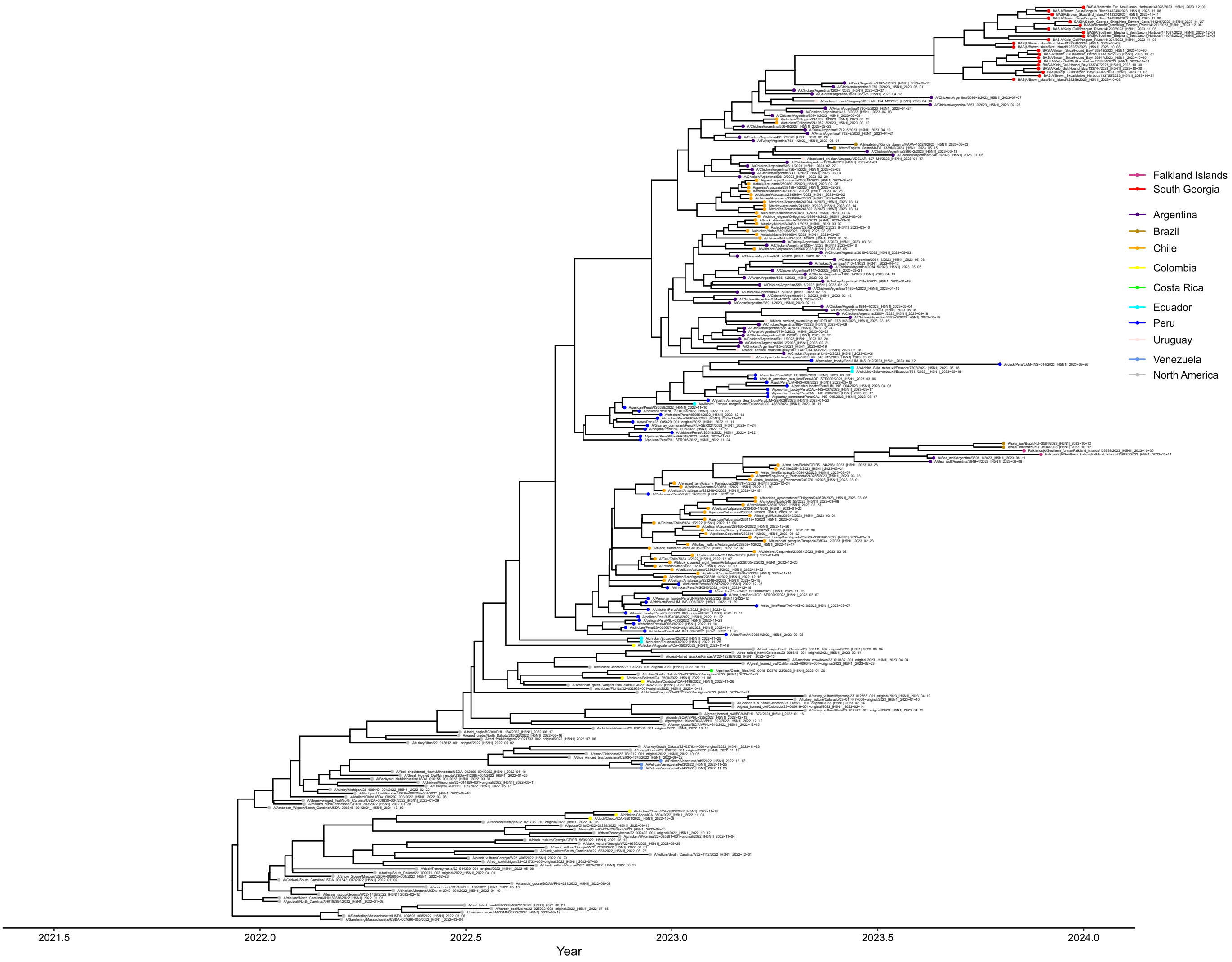

Supplementary Table 1: Summary of date, location and species of avian samples collected, and testing result for H5N1 HPAIV from South Georgia and the Falklands Islands. a, RRT-PCR interpretation from M-gene, H5-HP and N1; b, virus isolation in embryonated fowl eggs; c, oropharyngeal (OP); d, cloacal (C); e, not performed (N/P); f, not applicable (N/A); g, sequence derived from egg amplified material; h, performed but could not be determined (ND); -, No Cq.

Location	Collection date	Common name	Scientific name	Swab type	RRT-PCR assay			RRT-PCR Interpretation[a]	Virus isolation[b]	Virus strain name	NCBI Accession numbers sequential across segments (GISAID accession number)
					M-gene	H5-HP	N1				
Bird Island, South Georgia	08/10/2023	Brown skua	*Stercorarius antarcticus*	OP[c]	28.46	27.75	29.82	**H5N1 HPAIV**	Negative	A/brown_skua/Bird_Island/128287/2023	PQ129499- PQ129506 (EPI_ISL_18439562)
				C[d]	27.1	26.2	28.3	**H5N1 HPAIV**	Negative	N/P[e]	N/A[f]
		Brown skua	*Stercorarius antarcticus*	OP	28.09	26.25	28.99	**H5N1 HPAIV**	Negative	A/brown_skua/Bird_Island/128288/2023	PQ129515- PQ129522 (EPI_ISL_18439563)
				C	35.01	33.65	35.69	**H5N1 HPAIV**	Negative	N/P	N/A
		Brown skua	*Stercorarius antarcticus*	OP	31.07	30.16	32.36	**H5N1 HPAIV**	Negative	A/brown_skua/Bird_Island/128289/2023	PQ129507- PQ129514 (EPI_ISL_18439564)
				C	32.63	31.85	33.87	**H5N1 HPAIV**	Negative	N/P	N/A
	11/11/2023	Brown skua	*Stercorarius antarcticus*	OP	28.91	27.91	28.37	**H5N1 HPAIV**	Negative	A/brown_skua/Bird_Island/141232/2023	PQ114066-PQ114073 (EPI_ISL_18742214)
				C	-	-	38.04	Negative	Negative	N/P	N/A
Hound Bay, South Georgia	30/10/2023	Kelp gull	*Larus dominicanus*	C	-	-	-	Negative	N/P	N/P	N/A
				OP	27.27	25.24	27.96	**H5N1 HPAIV**	Negative	A/kelp_gull/Hound_Bay/133744/2023	PQ114082-PQ114089 (EPI_ISL_18592422)
		Brown skua	*Stercorarius antarcticus*	C	32.36	29.52	32.75	**H5N1 HPAIV**	**Positive**	A/brown_skua/Hound_Bay/133947/2023	PQ114034 -PQ114041 (EPI_ISL_18592428)
				OP	32.31	29.87	32.09	**H5N1 HPAIV**	N/P	N/P	N/A
		Kelp gull	*Larus dominicanus*	C	-	-	-	Negative	N/P	N/P	N/A
				OP	35.89	34.71	35.8	**H5N1 HPAIV**	Negative	N/P	N/A
		Kelp gull	*Larus dominicanus*	C	-	-	37.81	Negative	N/P	N/P	N/A
				OP	28.18	26.27	29.22	**H5N1 HPAIV**	**Positive**	A/kelp_gull/Hound_Bay/133747/2023	PQ114074-PQ114081 (EPI_ISL_18592423)
		Brown skua	*Stercorarius antarcticus*	C	28.86	26.87	29.88	**H5N1 HPAIV**	Negative	N/P	N/A
				OP	29.61	27.84	30.53	**H5N1 HPAIV**	N/P	N/P	N/A
		Brown skua	*Stercorarius antarcticus*	C	26.92	24.65	27.28	**H5N1 HPAIV**	**Positive**	A/brown_skua/Hound_Bay/133949/2023	PQ114018-PQ114025 (EPI_ISL_18592429)
				OP	27.61	27.39	29.47	**H5N1 HPAIV**	N/P	N/P	N/A
				C	36.31	35.76	36.67	**H5N1 HPAIV**	N/P	N/P	N/A

St Andrew's Bay, South Georgia		Brown skua	*Stercorarius antarcticus*	OP	29.34	26.5	28.64	**H5N1 HPAIV**	Negative	N/P	N/A
		Brown skua	*Stercorarius antarcticus*	C	32.43	30.89	33.86	**H5N1 HPAIV**	Negative	N/P	N/A
				OP	34.98	31.47	34.54	**H5N1 HPAIV**	N/P	N/P	N/A
Moltke Harbour, South Georgia	31/10/2023	Brown skua	*Stercorarius antarcticus*	C	-	-	-	Negative	N/P	N/P	N/A
				OP	31.43	28.42	31.77	**H5N1 HPAIV**	Negative	A/brown_skua/Moltke_Harbour/133752/2023	PQ113986-PQ113993 (EPI_ISL_18592424)
		Kelp gull	*Larus dominicanus*	C	-	-	-	Negative	N/P	N/P	N/A
				OP	36.29	33.65	35.55	**H5N1 HPAIV**	Negative	N/P	N/A
		Kelp gull	*Larus dominicanus*	C	38.84	-	39.19	Negative	N/P	N/P	N/A
				OP	29.21	27.54	29.78	**H5N1 HPAIV**	**Positive**	A/kelp_gull/Moltke_Harbour/133754/2023	PQ113978-PQ113985 (EPI_ISL_18592425)
		Brown skua	*Stercorarius antarcticus*	C	30.65	28.43	31.7	**H5N1 HPAIV**	N/P	N/P	N/A
				OP	27.25	25.51	28.42	**H5N1 HPAIV**	**Positive**	A/brown_skua/Moltke_Harbour/133755/2023	PQ113970-PQ113977 (EPI_ISL_18592426)
		Brown skua	*Stercorarius antarcticus*	C	32.66	30.37	33.76	**H5N1 HPAIV**	N/P	N/P	N/A
				OP	30.87	29.32	31.65	**H5N1 HPAIV**	Negative	N/P	N/A
		Kelp gull	*Larus dominicanus*	C	-	-	-	Negative	N/P	N/P	N/A
				OP	29.97	28.92	31.5	**H5N1 HPAIV**	Negative	N/P	N/A
Harpon Bay, South Georgia	03/11/2023	Kelp gull	*Larus dominicanus*	C	32.77	31.46	33.9	**H5N1 HPAIV**	N/P	N/P	N/A
				OP	30.67	28.51	31.04	**H5N1 HPAIV**	**Positive**	A/kelp_gull/Harpon_Bay/133943/2023	PQ113954-PQ113961 (EPI_ISL_18592427)
		Brown skua	*Stercorarius antarcticus*	C	30.09	29.26	31.53	**H5N1 HPAIV**	N/P	N/P	N/A
				OP	27.4	24.62	27.54	**H5N1 HPAIV**	Negative	N/P	N/A
		Kelp gull	*Larus dominicanus*	C	38.93	-	37.63	Negative	N/P	N/P	N/A
				OP	31.93	29.55	32.32	**H5N1 HPAIV**	Negative	N/P	N/A
		Kelp gull	*Larus dominicanus*	C	-	-	-	Negative	N/P	N/P	N/A
				OP	36.53	33.81	35.96	**H5N1 HPAIV**	Negative	N/P	N/A
Penguin River, South Georgia	08/11/2023	Kelp gull	*Larus dominicanus*	OP	27.88	26.95	27.32	**H5N1 HPAIV**	Negative	A/kelp_gull/Penguin_River/141234/2023	PQ114058-PQ114085 (EPI_ISL_18742215)
				C	-	-	-	Negative	N/P	N/P	N/A
		Brown skua	*Stercorarius antarcticus*	OP	27.96	26.77	28.27	**H5N1 HPAIV**	Negative	A/brown_skua/Penguin_River/141236/2023	PQ114002-PQ114009 (EPI_ISL_18742216)
				C	35.95	35.41	35.53	**H5N1 HPAIV**	N/P	N/P	N/A
		Kelp gull		OP	34.41	33.4	34.16	**H5N1 HPAIV**	N/P	N/P	N/A

			Larus dominicanus	C	30.31	29.69	29.92	**H5N1 HPAIV**	Negative	A/kelp_gull/Penguin_River/141239/2023	PQ113994-PQ114001 (EPI_ISL_18742217)
		Brown skua	*Stercorarius antarcticus*	OP	31.95	31.25	37.48	**H5N1 HPAIV**	Negative	A/brown_skua/Penguin_River/141240/2023	PQ114026-PQ114033 (EPI_ISL_18742218)
				C	36.65	28.5	29.27	**H5N1 HPAIV**	Negative	N/P	N/A
King Edward Cove, South Georgia	25/11/2023	South Georgia shag	*Leucocarbo georgianus*	OP	-	-	-	Negative	N/P	N/P	N/A
				C	-	-	-	Negative	N/P	N/P	N/A
	27/11/2023	South Georgia shag	*Leucocarbo georgianus*	OP	34.7	27.59	31.3	**H5N1 HPAIV**	N/P	N/P	N/A
				C	26.66	19.9	23.62	**H5N1 HPAIV**	**Positive**	A/South_Georgia_shag/King_Edward_Cove/141245/2023	PQ114010-PQ114017 (EPI_ISL_18742219)
	06/12/2023	South Georgia shag	*Leucocarbo georgianus*	OP	-	-	-	Negative	N/P	N/P	N/A
				C	-	-	-	Negative	N/P	N/P	N/A
		South Georgia shag	*Leucocarbo georgianus*	Brain	-	-	-	Negative	N/P	N/P	N/A
				OP	-	-	-	Negative	N/P	N/P	N/A
		South Georgia shag	*Leucocarbo georgianus*	C	-	-	-	Negative	N/P	N/P	N/A
				Brain	-	-	-	Negative	N/P	N/P	N/A
		King penguin	*Aptenodytes patagonicus*	OP	-	-	-	Negative	N/P	N/P	N/A
				C	-	-	-	Negative	N/P	N/P	N/A
				Brain	-	-	-	Negative	N/P	N/P	N/A
		Antarctic tern	*Sterna vittata*	OP	25.88	20.58	24.38	**H5N1 HPAIV**	Negative	N/P	N/A
				Brain	19.81	13.29	17.07	**H5N1 HPAIV**	Negative	A/ halacroc_tern/King_Edward_Point/141271/2023	PQ113962-PQ113969 (EPI_ISL_18742220)
Stanley, Falkland Islands	30/10/2023	Southern fulmar	*Fulmarus glacialoides*	OP	20.5	18.65	20.71	**H5N1 HPAIV**	Negative	A/Southern_fulmar/Falkland_Island/133789/2023	PQ113930-PQ113937 (EPI_ISL_18522961)
				C	18.85	17.57	19.62	**H5N1 HPAIV**	Negative	N/P	N/A
				Brain	21.51	20.49	22.41	**H5N1 HPAIV**	**Positive**	N/P	N/A
	06/11/2023	Grey-backed storm petrel	*Garrodia nereis*	OP	39.83	-	-	Negative	N/P	N/P	N/A
				C	-	-	-	Negative	N/P	N/P	N/A
		Falkland steamer duck	*Tachyeres brachypterus*	OP	39.37	-	-	Negative	N/P	N/P	N/A
				C	-	-	-	Negative	N/P	N/P	N/A
	13/11/2023	Southern fulmar	*Fulmarus glacialoides*	OP	28.81	25.58	26.85	**H5N1 HPAIV**	**Positive**	A/Southern_fulmar/Falkland_Island/138870/2023[g]	PQ113938-PQ113945 (EPI_ISL_18918640)
				C	32.89	29.45	30.98	**H5N1 HPAIV**	Negative	N/P	N/A
	24/11/2023			OP	-	-	-	Negative	N/P	N/P	N/A

		Austral thrush	*Turdus falcklandii*	C	-	-	-	Negative	N/P	N/P	N/A
	25/11/2023	Rock shag	*halacrocorax magellanicus*	OP	-	-	-	Negative	N/P	N/P	N/A
				C	-	-	-	Negative	N/P	N/P	N/A
	27/11/2023	Chicken	*Gallus gallus domesticus*	OP	-	-	-	Negative	N/P	N/P	N/A
				C	-	-	-	Negative	N/P	N/P	N/A
Saunders Island, Falkland Islands	19/11/2023	Black browed albatross	*Thalassarche melanophris*	OP	36.51	33.92	35.84	**H5N1 HPAIV**	Negative	N/P	N/A
				C	31.61	29.46	30.81	**H5N1 HPAIV**	Negative	N/D[h]	N/A
Steeple Jason, Falkland Islands	26/11/2023	Black browed albatross	*Thalassarche melanophris*	OP	-	-	-	Negative	N/P	N/P	N/A
				C	-	-	-	Negative	N/P	N/P	N/A
		Black browed albatross	*Thalassarche melanophris*	OP	34.75	34.6	32.87	**H5N1 HPAIV**	Negative	N/P	N/A
				C	30.47	30.94	29.16	**H5N1 HPAIV**	Negative	N/D	N/A
New Island, Falkland Islands	01/12/2023	Imperial shag	*Leucocarbo atriceps*	OP	-	-	-	Negative	N/P	N/P	N/A
				C	-	-	-	Negative	N/P	N/P	N/A
	04/12/2023	Black browed albatross	*Thalassarche melanophris*	C	-	-	-	Negative	N/P	N/P	N/A
				OP	-	-	-	Negative	N/P	N/P	N/A
Kidney Island, Falkland Islands	10/12/2023	Southern rockhopper penguin	*Eudyptes chrysocome*	C	-	-	-	Negative	N/P	N/P	N/A
				OP	-	-	-	Negative	N/P	N/P	N/A

Supplementary Table 2: Summary of date, location and species of mammalian samples collected and testing result for H5 HPAIV from South Georgia. A, RRT-PCR interpretation from M-gene, H5-HP and N1; b, virus isolation in embryonated fowl eggs; c, not performed (N/P); d, not applicable (N/A); f, performed but could not be determined (ND); -, No Cq

Location	Collection date	Age	Common name	Scientific name	Swab type	RRT-PCR assay			RRT-PCR Interpretation[a]	Virus isolation[b]	Virus strain name	GISAID accession number
						M-gene	H5-HP	N1				
Moltke Harbour, South Georgia	31/10/2023	Pup	Southern elephant seal	*Mirounga leonina*	Nasal	37.75	-	39.5	Negative	Negative	N/P[c]	N/A[d]
					Anal	-	-	-	Negative	N/P	N/P	N/A
			Southern elephant seal	*Mirounga leonina*	Nasal	-	-	-	Negative	N/P	N/P	N/A
					Anal	-	-	-	Negative	N/P	N/P	N/A
			Southern elephant seal	*Mirounga leonina*	Nasal	38.8	-	-	Negative	Negative	N/P	N/A
					Anal	-	-	-	Negative	N/P	N/P	N/A
King Edward Cove, South Georgia	06/12/2023	Pup (newborn)	Antarctic fur seal	*Arctocephalus gazella*	Oral	-	-	-	Negative	N/P	N/P	N/A
					Nasal	-	-	-	Negative	N/P	N/P	N/A
					Anal	-	-	-	Negative	N/P	N/P	N/A
					Brain	-	-	-	Negative	N/P	N/P	N/A
)Jason Harbour, South Georgia	09/12/2023	Weaner (molting)	Southern elephant seal	*Mirounga leonina*	Oral	37.21	34.44	36.58	**H5N1 HPAIV**	Negative	A/Southern_elephant_seal/Jason_Harbour/141027/2023	PQ113946-PQ113953 (EPI_ISL_18742221)
					Anal	-	-	-	Negative	N/P	N/P	N/A
					Nasal	-	-	39.93	Negative	N/P	N/P	N/A
					Brain	-	-	-	Negative	N/P	N/P	N/A
			Southern elephant seal	*Mirounga leonina*	Oral	-	-	-	Negative	N/P	N/P	N/A
					Anal	-	-	-	Negative	N/P	N/P	N/A
					Nasal	-	-	-	Negative	N/P	N/P	N/A
					Brain	-	-	-	Negative	N/P	N/P	N/A
			Southern elephant seal	*Mirounga leonina*	Brain	-	-	-	Negative	N/P	N/P	N/A
			Southern elephant seal	*Mirounga leonina*	Oral	-	-	-	Negative	N/P	N/P	N/A
					Anal	-	-	-	Negative	N/P	N/P	N/A
					Nasal	-	-	-	Negative	N/P	N/P	N/A

					Brain	-	-	-	Negative	N/P	N/P	N/A
			Southern elephant seal	*Mirounga leonina*	Oral	-	-	-	Negative	N/P	N/P	N/A
					Anal	-	-	-	Negative	N/P	N/P	N/A
					Nasal	-	-	37.97	Negative	N/P	N/P	N/A
					Brain	-	-	-	Negative	N/P	N/P	N/A
			Southern elephant seal	*Mirounga leonina*	Oral	-	-	-	Negative	N/P	N/P	N/A
					Anal	32.32	30.47	32.09	**H5N1 HPAIV**	Negative	N/D[f]	N/A
					Nasal	38.27	-	39.31	Negative	N/P	N/P	N/A
					Brain	-	-	-	Negative	N/P	N/P	N/A
			Southern elephant seal	*Mirounga leonina*	Oral	37.84	-	38.38	Negative	N/P	N/P	N/A
					Anal	35.51	34.25	34.21	**H5N1 HPAIV**	Negative	N/D	N/A
					Nasal	-	-	37.88	Negative	N/P	N/P	N/A
					Brain	33.35	32.84	33.2	**H5N1 HPAIV**	Negative	N/D	N/A
			Southern elephant seal	*Mirounga leonina*	Oral	-	-	-	Negative	N/P	N/P	N/A
					Anal	-	-	-	Negative	N/P	N/P	N/A
					Nasal	-	-	-	Negative	N/P	N/P	N/A
					Brain	-	-	-	Negative	N/P	N/P	N/A
			Southern elephant seal	*Mirounga leonina*	Oral	-	-	39.86	Negative	N/P	N/P	N/A
					Anal	-	-	-	Negative	N/P	N/P	N/A
					Nasal	-	-	-	Negative	N/P	N/P	N/A
					Brain	32.78	31.02	32.63	**H5N1 HPAIV**	Negative	N/D	N/A
			Southern elephant seal	*Mirounga leonina*	Oral	-	-	39.64	Negative	N/P	N/P	N/A
					Anal	37.98	-	36.14	Negative	N/P	N/P	N/A
					Nasal	36.05	35.1	36.23	**H5N1 HPAIV**	Negative	N/D	N/A
					Brain	37.04	-	39.35	Negative	N/P	N/P	N/A
					Liver	-	-	-	Negative	N/P	N/P	N/A
					Lung	32.03	31.41	32.24	**H5N1 HPAIV**	Negative	A/Southern_elephant_seal/Jason_Harbour/141078/2023	PQ114042-PQ114049 (EPI_ISL_18742213)
					Blood	-	-	-	Negative	N/P	N/P	N/A

				Antarctic fur seal	*Arctocephalus gazella*	Oral	-	-	-	Negative	N/P	N/P	N/A
						Anal	-	-	-	Negative	N/P	N/P	N/A
						Nasal	36.9	-	-	Negative	N/P	N/P	N/A
						Brain	-	-	-	Negative	N/P	N/P	N/A
				Antarctic fur seal	*Arctocephalus gazella*	Oral	30.66	29.87	31.18	**H5N1 HPAIV**	Negative	A/Antarctic_fur_seal/Jason_Harbour/141037/2023	PQ114050-PQ114057 (EPI_ISL_18742212)
			Adult male			Anal	-	-	-	Negative	N/P	N/P	N/A
						Nasal	32.41	33.81	35.49	**H5N1 HPAIV**	Negative	N/P	N/A
						Brain	31.61	33.71	32.59	**H5N1 HPAIV**	Negative	N/P	N/A
				Antarctic fur seal	*Arctocephalus gazella*	Oral	-	-	-	Negative	N/P	N/P	N/A
						Anal	-	-	-	Negative	N/P	N/P	N/A
						Nasal	-	-	-	Negative	N/P	N/P	N/A
						Brain	-	-	-	Negative	N/P	N/P	N/A

Supplementary Table 3: Percentage nucleotide (nt) identity across H5N1 HPAIV sequences generated from South Georgia and The Falkland Islands.

Segment	Sequence	A/Kelp Gull/Hound Bay/133744/2023	A/Kelp Gull/Hound Bay/133747/2023	A/Brown Skua/Moltke Harbour/133752/2023	A/Kelp Gull/Moltke Harbour/133754/2023	A/Brown Skua/Moltke Harbour/133755/2023	A/Kelp Gull/Harpon Bay/133943/2023	A/Brown Skua/Hound Bay/133947/2023	A/Brown Skua/Hound Bay/133949/2023	A/Brown skua/Bird Island/128289/2023	A/Brown skua/Bird Island/128288/2023	A/Brown skua/Bird Island/128287/2023	A/Kelp Gull/Penguin River/141239/2023	A/Brown Skua/Penguin River/141236/2023	A/Kelp Gull/Penguin River/141234/2023	A/Brown Skua/Bird Island/141232/2023	A/Southern Elephant Seal/Jason Harbour/141078/2023	A/Antarctic Fur Seal/Jason Harbour/141037/2023	A/Southern Elephant Seal/Jason Harbour/141027/2023	A/Southern Fulmar/Falkland Islands/138870/2023	A/Southern fulmar/Falkland Islands/133789/2023
PB2	A/Kelp Gull/Hound Bay/133744/2023	100	99.86	99.82	99.82	99.87	99.96	99.91	99.78	99.96	99.91	99.96	99.78	99.78	99.82	99.78	99.96	99.78	99.82	99.52	99.43
	A/Kelp Gull/Hound Bay/133747/2023	99.86	100	99.77	99.77	99.73	99.82	99.95	99.73	99.91	99.86	99.91	99.73	99.73	99.77	99.73	99.82	99.73	99.68	99.46	99.41
	A/Brown Skua/Moltke Harbour/133752/2023	99.82	99.77	100	99.82	99.69	99.78	99.82	99.96	99.87	99.82	99.87	99.69	99.69	99.74	99.69	99.78	99.69	99.65	99.43	99.34
	A/Kelp Gull/Moltke Harbour/133754/2023	99.82	99.77	99.82	100	99.69	99.78	99.82	99.78	99.87	99.82	99.87	99.69	99.69	99.74	99.69	99.78	99.69	99.65	99.43	99.34
	A/Brown Skua/Moltke Harbour/133755/2023	99.87	99.73	99.69	99.69	100	99.82	99.78	99.65	99.82	99.78	99.82	99.65	99.65	99.69	99.65	99.82	99.65	99.69	99.39	99.3
	A/Kelp Gull/Harpon Bay/133943/2023	99.96	99.82	99.78	99.78	99.82	100	99.87	99.74	99.91	99.87	99.91	99.74	99.74	99.78	99.74	99.91	99.74	99.78	99.56	99.47
	A/Brown Skua/Hound Bay/133947/2023	99.91	99.95	99.82	99.82	99.78	99.87	100	99.78	99.96	99.91	99.96	99.78	99.78	99.82	99.78	99.87	99.78	99.74	99.52	99.43
	A/Brown Skua/Hound Bay/133949/2023	99.78	99.73	99.96	99.78	99.65	99.74	99.78	100	99.82	99.78	99.82	99.65	99.65	99.69	99.65	99.74	99.65	99.61	99.39	99.3
	A/Brown skua/Bird Island/128289/2023	99.96	99.91	99.87	99.87	99.82	99.91	99.96	99.82	100	99.96	100	99.82	99.82	99.87	99.82	99.91	99.82	99.78	99.56	99.47
	A/Brown skua/Bird Island/128288/2023	99.91	99.86	99.82	99.82	99.78	99.87	99.91	99.78	99.96	100	99.96	99.78	99.78	99.82	99.78	99.87	99.78	99.74	99.52	99.43
	A/Brown skua/Bird Island/128287/2023	99.96	99.91	99.87	99.87	99.82	99.91	99.96	99.82	100	99.96	100	99.82	99.82	99.87	99.82	99.91	99.82	99.78	99.56	99.47
	A/Kelp Gull/Penguin River/141239/2023	99.78	99.73	99.69	99.69	99.65	99.74	99.78	99.65	99.82	99.78	99.82	100	100	99.96	100	99.74	100	99.96	99.39	99.3
	A/Brown Skua/Penguin River/141236/2023	99.78	99.73	99.69	99.69	99.65	99.74	99.78	99.65	99.82	99.78	99.82	100	100	99.96	100	99.74	100	99.96	99.39	99.3
	A/Kelp Gull/Penguin River/141234/2023	99.82	99.77	99.74	99.74	99.69	99.78	99.82	99.69	99.87	99.82	99.87	99.96	99.96	100	99.96	99.78	99.96	99.91	99.43	99.34
	A/Brown Skua/Bird Island/141232/2023	99.78	99.73	99.69	99.69	99.65	99.74	99.78	99.65	99.82	99.78	99.82	100	100	99.96	100	99.74	100	99.96	99.39	99.3
	A/Southern Elephant Seal/Jason Harbour/141078/2023	99.96	99.82	99.78	99.78	99.82	99.91	99.87	99.74	99.91	99.87	99.91	99.74	99.74	99.78	99.74	100	99.74	99.78	99.47	99.39
	A/Antarctic Fur Seal/Jason Harbour/141037/2023	99.78	99.73	99.69	99.69	99.65	99.74	99.78	99.65	99.82	99.78	99.82	100	100	99.96	100	99.74	100	99.96	99.39	99.3
	A/Southern Elephant Seal/Jason Harbour/141027/2023	99.82	99.68	99.65	99.65	99.69	99.78	99.74	99.61	99.78	99.74	99.78	99.96	99.96	99.91	99.96	99.78	99.96	100	99.34	99.25
	A/Southern Fulmar/Falkland Islands/138870/2023	99.52	99.46	99.43	99.43	99.39	99.56	99.52	99.39	99.56	99.52	99.56	99.39	99.39	99.43	99.39	99.47	99.39	99.34	100	99.91
	A/Southern fulmar/Falkland Islands/133789/2023	99.43	99.41	99.34	99.34	99.3	99.47	99.43	99.3	99.47	99.43	99.47	99.3	99.3	99.34	99.3	99.39	99.3	99.25	99.91	100
PB1	A/Kelp Gull/Hound Bay/133744/2023	100	99.82	99.91	99.82	99.82	99.91	99.91	99.91	99.78	99.87	99.87	99.78	99.78	99.87	99.78	99.91	99.78	99.91	99.34	99.43
	A/Kelp Gull/Hound Bay/133747/2023	99.82	100	99.86	99.77	99.72	99.82	99.91	99.82	99.68	99.77	99.77	99.68	99.68	99.77	99.68	99.82	99.68	99.82	99.36	99.45
	A/Brown Skua/Moltke Harbour/133752/2023	99.91	99.86	100	99.91	99.82	99.91	99.91	100	99.86	99.95	99.95	99.86	99.86	99.95	99.86	99.91	99.86	99.91	99.37	99.46
	A/Kelp Gull/Moltke Harbour/133754/2023	99.82	99.77	99.91	100	99.73	99.82	99.82	99.91	99.77	99.86	99.86	99.77	99.77	99.86	99.77	99.82	99.77	99.82	99.28	99.37
	A/Brown Skua/Moltke Harbour/133755/2023	99.82	99.72	99.82	99.73	100	99.82	99.82	99.82	99.69	99.78	99.78	99.69	99.69	99.78	99.69	99.82	99.69	99.82	99.29	99.38
	A/Kelp Gull/Harpon Bay/133943/2023	99.91	99.82	99.91	99.82	99.82	100	99.91	99.91	99.78	99.87	99.87	99.78	99.78	99.87	99.78	99.91	99.78	99.91	99.34	99.43
	A/Brown Skua/Hound Bay/133947/2023	99.91	99.91	99.91	99.82	99.82	99.91	100	99.91	99.78	99.87	99.87	99.78	99.78	99.87	99.78	99.91	99.78	99.91	99.34	99.43
	A/Brown Skua/Hound Bay/133949/2023	99.91	99.82	100	99.91	99.82	99.91	99.91	100	99.87	99.96	99.96	99.87	99.87	99.96	99.87	99.91	99.87	99.91	99.34	99.43
	A/Brown skua/Bird Island/128289/2023	99.78	99.68	99.86	99.77	99.69	99.78	99.78	99.87	100	99.91	99.91	99.74	99.74	99.82	99.74	99.78	99.74	99.78	99.21	99.3
	A/Brown skua/Bird Island/128288/2023	99.87	99.77	99.95	99.86	99.78	99.87	99.87	99.96	99.91	100	100	99.82	99.82	99.91	99.82	99.87	99.82	99.87	99.3	99.38
	A/Brown skua/Bird Island/128287/2023	99.87	99.77	99.95	99.86	99.78	99.87	99.87	99.96	99.91	100	100	99.82	99.82	99.91	99.82	99.87	99.82	99.87	99.3	99.38
	A/Kelp Gull/Penguin River/141239/2023	99.78	99.68	99.86	99.77	99.69	99.78	99.78	99.87	99.74	99.82	99.82	100	100	99.91	100	99.78	100	99.87	99.21	99.3

	A/Brown Skua/Penguin River/141236/2023	99.78	99.68	99.86	99.77	99.69	99.78	99.78	99.87	99.74	99.82	99.82	100	100	99.91	100	99.78	100	99.87	99.21	99.3
	A/Kelp Gull/Penguin River/141234/2023	99.87	99.77	99.95	99.86	99.78	99.87	99.87	99.96	99.82	99.91	99.91	99.91	99.91	100	99.91	99.87	99.91	99.87	99.3	99.38
	A/Brown Skua/Bird Island/141232/2023	99.78	99.68	99.86	99.77	99.69	99.78	99.78	99.87	99.74	99.82	99.82	100	100	99.91	100	99.78	100	99.87	99.21	99.3
	A/Southern Elephant Seal/Jason Harbour/141078/2023	99.91	99.82	99.91	99.82	99.82	99.91	99.91	99.91	99.78	99.87	99.87	99.78	99.78	99.87	99.78	100	99.78	99.91	99.34	99.43
	A/Antarctic Fur Seal/Jason Harbour/141037/2023	99.78	99.68	99.86	99.77	99.69	99.78	99.78	99.87	99.74	99.82	99.82	100	100	99.91	100	99.78	100	99.87	99.21	99.3
	A/Southern Elephant Seal/Jason Harbour/141027/2023	99.91	99.82	99.91	99.82	99.82	99.91	99.91	99.91	99.78	99.87	99.87	99.87	99.87	99.87	99.87	99.91	99.87	100	99.34	99.43
	A/Southern Fulmar/Falkland Islands/138870/2023	99.34	99.36	99.37	99.28	99.29	99.34	99.34	99.34	99.21	99.3	99.3	99.21	99.21	99.3	99.21	99.34	99.21	99.34	100	99.82
	A/Southern fulmar/Falkland Islands/133789/2023	99.43	99.45	99.46	99.37	99.38	99.43	99.43	99.43	99.3	99.38	99.38	99.3	99.3	99.38	99.3	99.43	99.3	99.43	99.82	100
PA	A/Kelp Gull/Hound Bay/133744/2023	100	99.77	99.72	99.72	99.77	100	99.77	99.77	99.86	99.86	99.86	99.81	99.81	99.81	99.81	99.81	99.81	99.67	98.98	98.84
PA	A/Kelp Gull/Hound Bay/133747/2023	99.77	100	99.77	99.77	99.63	99.77	99.81	99.81	99.91	99.91	99.91	99.86	99.86	99.86	99.86	99.77	99.86	99.72	99.02	98.88
PA	A/Brown Skua/Moltke Harbour/133752/2023	99.72	99.77	100	99.72	99.58	99.72	99.77	99.86	99.86	99.86	99.86	99.81	99.81	99.81	99.81	99.72	99.81	99.67	98.98	98.84
PA	A/Kelp Gull/Moltke Harbour/133754/2023	99.72	99.77	99.72	100	99.63	99.72	99.77	99.77	99.86	99.86	99.86	99.81	99.81	99.81	99.81	99.72	99.81	99.67	98.97	98.83
PA	A/Brown Skua/Moltke Harbour/133755/2023	99.77	99.63	99.58	99.63	100	99.77	99.63	99.63	99.72	99.72	99.72	99.67	99.67	99.67	99.67	99.67	99.67	99.53	98.84	98.7
PA	A/Kelp Gull/Harpon Bay/133943/2023	100	99.77	99.72	99.72	99.77	100	99.77	99.77	99.86	99.86	99.86	99.81	99.81	99.81	99.81	99.81	99.81	99.67	98.98	98.84
PA	A/Brown Skua/Hound Bay/133947/2023	99.77	99.81	99.77	99.77	99.63	99.77	100	99.81	99.91	99.91	99.91	99.86	99.86	99.86	99.86	99.77	99.86	99.72	99.02	98.88
PA	A/Brown Skua/Hound Bay/133949/2023	99.77	99.81	99.86	99.77	99.63	99.77	99.81	100	99.91	99.91	99.91	99.86	99.86	99.86	99.86	99.77	99.86	99.72	99.02	98.88
PA	A/Brown skua/Bird Island/128289/2023	99.86	99.91	99.86	99.86	99.72	99.86	99.91	99.91	100	100	100	99.95	99.95	99.95	99.95	99.86	99.95	99.81	99.12	98.98
PA	A/Brown skua/Bird Island/128288/2023	99.86	99.91	99.86	99.86	99.72	99.86	99.91	99.91	100	100	100	99.95	99.95	99.95	99.95	99.86	99.95	99.81	99.12	98.98
PA	A/Brown skua/Bird Island/128287/2023	99.86	99.91	99.86	99.86	99.72	99.86	99.91	99.91	100	100	100	99.95	99.95	99.95	99.95	99.86	99.95	99.81	99.12	98.98
PA	A/Kelp Gull/Penguin River/141239/2023	99.81	99.86	99.81	99.81	99.67	99.81	99.86	99.86	99.95	99.95	99.95	100	100	100	100	99.81	100	99.77	99.07	98.93
PA	A/Brown Skua/Penguin River/141236/2023	99.81	99.86	99.81	99.81	99.67	99.81	99.86	99.86	99.95	99.95	99.95	100	100	100	100	99.81	100	99.77	99.07	98.93
PA	A/Kelp Gull/Penguin River/141234/2023	99.81	99.86	99.81	99.81	99.67	99.81	99.86	99.86	99.95	99.95	99.95	100	100	100	100	99.81	100	99.77	99.07	98.93
PA	A/Brown Skua/Bird Island/141232/2023	99.81	99.86	99.81	99.81	99.67	99.81	99.86	99.86	99.95	99.95	99.95	100	100	100	100	99.81	100	99.77	99.07	98.93
PA	A/Southern Elephant Seal/Jason Harbour/141078/2023	99.81	99.77	99.72	99.72	99.67	99.81	99.77	99.77	99.86	99.86	99.86	99.81	99.81	99.81	99.81	100	99.81	99.77	98.98	98.84
PA	A/Antarctic Fur Seal/Jason Harbour/141037/2023	99.81	99.86	99.81	99.81	99.67	99.81	99.86	99.86	99.95	99.95	99.95	100	100	100	100	99.81	100	99.77	99.07	98.93
PA	A/Southern Elephant Seal/Jason Harbour/141027/2023	99.67	99.72	99.67	99.67	99.53	99.67	99.72	99.72	99.81	99.81	99.81	99.77	99.77	99.77	99.77	99.77	99.77	100	98.93	98.79
PA	A/Southern Fulmar/Falkland Islands/138870/2023	98.98	99.02	98.98	98.97	98.84	98.98	99.02	99.02	99.12	99.12	99.12	99.07	99.07	99.07	99.07	98.98	99.07	98.93	100	99.77
PA	A/Southern fulmar/Falkland Islands/133789/2023	98.84	98.88	98.84	98.83	98.7	98.84	98.88	98.88	98.98	98.98	98.98	98.93	98.93	98.93	98.93	98.84	98.93	98.79	99.77	100
HA	A/Kelp Gull/Hound Bay/133744/2023	100	99.77	99.88	99.77	99.94	99.94	99.88	99.88	99.88	99.77	99.82	99.71	99.53	99.77	99.47	99.71	99.47	99.71	99	99.06
HA	A/Kelp Gull/Hound Bay/133747/2023	99.77	100	99.88	99.77	99.82	99.82	99.88	99.88	99.88	99.77	99.82	99.71	99.53	99.77	99.47	99.59	99.47	99.71	99	99.06
HA	A/Brown Skua/Moltke Harbour/133752/2023	99.88	99.88	100	99.88	99.94	99.94	100	100	100	99.88	99.94	99.88	99.71	99.88	99.65	99.71	99.65	99.88	99.12	99.18
HA	A/Kelp Gull/Moltke Harbour/133754/2023	99.77	99.77	99.88	100	99.82	99.82	99.88	99.88	99.88	99.77	99.82	99.71	99.53	99.77	99.47	99.59	99.47	99.71	99	99.06
HA	A/Brown Skua/Moltke Harbour/133755/2023	99.94	99.82	99.94	99.82	100	100	99.94	99.94	99.94	99.82	99.88	99.77	99.59	99.82	99.53	99.77	99.53	99.76	99.06	99.12
HA	A/Kelp Gull/Harpon Bay/133943/2023	99.94	99.82	99.94	99.82	100	100	99.94	99.94	99.94	99.82	99.88	99.77	99.59	99.82	99.53	99.77	99.53	99.77	99.06	99.12
HA	A/Brown Skua/Hound Bay/133947/2023	99.88	99.88	100	99.88	99.94	99.94	100	100	100	99.88	99.94	99.82	99.65	99.88	99.59	99.71	99.59	99.82	99.12	99.18
HA	A/Brown Skua/Hound Bay/133949/2023	99.88	99.88	100	99.88	99.94	99.94	100	100	100	99.88	99.94	99.82	99.65	99.88	99.59	99.71	99.59	99.82	99.12	99.18
HA	A/Brown skua/Bird Island/128289/2023	99.88	99.88	100	99.88	99.94	99.94	100	100	100	99.88	99.94	99.82	99.65	99.88	99.59	99.71	99.59	99.82	99.12	99.18
HA	A/Brown skua/Bird Island/128288/2023	99.77	99.77	99.88	99.77	99.82	99.82	99.88	99.88	99.88	100	99.94	99.82	99.65	99.88	99.59	99.71	99.59	99.88	99	99.18
HA	A/Brown skua/Bird Island/128287/2023	99.82	99.82	99.94	99.82	99.88	99.88	99.94	99.94	99.94	99.94	100	99.88	99.71	99.94	99.65	99.77	99.65	99.88	99.06	99.24
HA	A/Kelp Gull/Penguin River/141239/2023	99.71	99.71	99.88	99.71	99.77	99.77	99.82	99.82	99.82	99.82	99.88	100	99.82	99.82	99.65	99.65	99.65	99.88	98.94	99.12
HA	A/Brown Skua/Penguin River/141236/2023	99.53	99.53	99.71	99.53	99.59	99.59	99.65	99.65	99.65	99.65	99.71	99.82	100	99.65	99.82	99.47	99.82	99.82	98.77	98.94
HA	A/Kelp Gull/Penguin River/141234/2023	99.77	99.77	99.88	99.77	99.82	99.82	99.88	99.88	99.88	99.88	99.94	99.82	99.65	100	99.59	99.71	99.71	99.82	99.12	99.3
HA	A/Brown Skua/Bird Island/141232/2023	99.47	99.47	99.65	99.47	99.53	99.53	99.59	99.59	99.59	99.59	99.65	99.65	99.82	99.59	100	99.41	99.88	99.77	98.71	98.88
HA	A/Southern Elephant Seal/Jason Harbour/141078/2023	99.71	99.59	99.71	99.59	99.77	99.77	99.71	99.71	99.71	99.71	99.77	99.65	99.47	99.71	99.41	100	99.41	99.65	98.83	99
HA	A/Antarctic Fur Seal/Jason Harbour/141037/2023	99.47	99.47	99.65	99.47	99.53	99.53	99.59	99.59	99.59	99.59	99.65	99.65	99.82	99.71	99.88	99.41	100	99.77	98.83	99
HA	A/Southern Elephant Seal/Jason Harbour/141027/2023	99.71	99.71	99.88	99.71	99.76	99.77	99.82	99.82	99.82	99.88	99.88	99.88	99.82	99.82	99.77	99.65	99.77	100	98.94	99.12
HA	A/Southern Fulmar/Falkland Islands/138870/2023	99	99	99.12	99	99.06	99.06	99.12	99.12	99.12	99	99.06	98.94	98.77	99.12	98.71	98.83	98.83	98.94	100	99.59
HA	A/Southern fulmar/Falkland Islands/133789/2023	99.06	99.06	99.18	99.06	99.12	99.12	99.18	99.18	99.18	99.18	99.24	99.12	98.94	99.3	98.88	99	99	99.12	99.59	100
NP	A/Kelp Gull/Hound Bay/133744/2023	100	99.8	99.67	99.86	99.93	99.93	99.6	99.93	99.93	99.93	99.93	99.93	99.93	99.93	99.93	99.87	99.93	99.8	99.06	99.13

	A/Kelp Gull/Hound Bay/133747/2023	99.8	100	99.6	99.8	99.87	99.87	99.67	99.87	99.87	99.87	99.87	99.87	99.87	99.87	99.87	99.8	99.87	99.73	99	99.06
	A/Brown Skua/Moltke Harbour/133752/2023	99.67	99.6	100	99.8	99.73	99.6	99.4	99.73	99.73	99.73	99.73	99.73	99.73	99.73	99.73	99.67	99.73	99.6	98.86	98.93
	A/Kelp Gull/Moltke Harbour/133754/2023	99.86	99.8	99.8	100	99.93	99.8	99.59	99.93	99.93	99.93	99.93	99.93	99.93	99.93	99.93	99.86	99.93	99.8	99.05	99.12
	A/Brown Skua/Moltke Harbour/133755/2023	99.93	99.87	99.73	99.93	100	99.87	99.67	100	100	100	100	100	100	100	100	99.93	100	99.87	99.13	99.2
	A/Kelp Gull/Harpon Bay/133943/2023	99.93	99.87	99.6	99.8	99.87	100	99.67	99.87	99.87	99.87	99.87	99.87	99.87	99.87	99.87	99.8	99.87	99.73	99	99.06
	A/Brown Skua/Hound Bay/133947/2023	99.6	99.67	99.4	99.59	99.67	99.67	100	99.67	99.67	99.67	99.67	99.67	99.67	99.67	99.67	99.6	99.67	99.53	98.8	98.86
	A/Brown Skua/Hound Bay/133949/2023	99.93	99.87	99.73	99.93	100	99.87	99.67	100	100	100	100	100	100	100	100	99.93	100	99.87	99.13	99.2
	A/Brown skua/Bird Island/128289/2023	99.93	99.87	99.73	99.93	100	99.87	99.67	100	100	100	100	100	100	100	100	99.93	100	99.87	99.13	99.2
	A/Brown skua/Bird Island/128288/2023	99.93	99.87	99.73	99.93	100	99.87	99.67	100	100	100	100	100	100	100	100	99.93	100	99.87	99.13	99.2
	A/Brown skua/Bird Island/128287/2023	99.93	99.87	99.73	99.93	100	99.87	99.67	100	100	100	100	100	100	100	100	99.93	100	99.87	99.13	99.2
	A/Kelp Gull/Penguin River/141239/2023	99.93	99.87	99.73	99.93	100	99.87	99.67	100	100	100	100	100	100	100	100	99.93	100	99.87	99.13	99.2
	A/Brown Skua/Penguin River/141236/2023	99.93	99.87	99.73	99.93	100	99.87	99.67	100	100	100	100	100	100	100	100	99.93	100	99.87	99.13	99.2
	A/Kelp Gull/Penguin River/141234/2023	99.93	99.87	99.73	99.93	100	99.87	99.67	100	100	100	100	100	100	100	100	99.93	100	99.87	99.13	99.2
	A/Brown Skua/Bird Island/141232/2023	99.93	99.87	99.73	99.93	100	99.87	99.67	100	100	100	100	100	100	100	100	99.93	100	99.87	99.13	99.2
	A/Southern Elephant Seal/Jason Harbour/141078/2023	99.87	99.8	99.67	99.86	99.93	99.8	99.6	99.93	99.93	99.93	99.93	99.93	99.93	99.93	99.93	100	99.93	99.93	99.06	99.13
	A/Antarctic Fur Seal/Jason Harbour/141037/2023	99.93	99.87	99.73	99.93	100	99.87	99.67	100	100	100	100	100	100	100	100	99.93	100	99.87	99.13	99.2
	A/Southern Elephant Seal/Jason Harbour/141027/2023	99.8	99.73	99.6	99.8	99.87	99.73	99.53	99.87	99.87	99.87	99.87	99.87	99.87	99.87	99.87	99.93	99.87	100	99	99.06
	A/Southern Fulmar/Falkland Islands/138870/2023	99.06	99	98.86	99.05	99.13	99	98.8	99.13	99.13	99.13	99.13	99.13	99.13	99.13	99.13	99.06	99.13	99	100	99.93
	A/Southern fulmar/Falkland Islands/133789/2023	99.13	99.06	98.93	99.12	99.2	99.06	98.86	99.2	99.2	99.2	99.2	99.2	99.2	99.2	99.2	99.13	99.2	99.06	99.93	100
NA	A/Kelp Gull/Hound Bay/133744/2023	100	99.79	99.72	99.79	99.65	99.79	99.79	99.72	99.57	99.57	99.57	99.72	99.72	99.79	99.72	99.79	99.72	99.79	99.08	99.01
	A/Kelp Gull/Hound Bay/133747/2023	99.79	100	99.93	100	99.72	99.72	100	99.93	99.79	99.79	99.79	99.93	99.93	100	99.93	100	99.93	100	99.29	99.22
	A/Brown Skua/Moltke Harbour/133752/2023	99.72	99.93	100	99.93	99.57	99.57	99.86	99.93	99.65	99.65	99.65	99.79	99.79	99.86	99.79	99.86	99.79	99.86	99.15	99.08
	A/Kelp Gull/Moltke Harbour/133754/2023	99.79	100	99.93	100	99.72	99.72	100	99.93	99.79	99.79	99.79	99.93	99.93	100	99.93	100	99.93	100	99.29	99.22
	A/Brown Skua/Moltke Harbour/133755/2023	99.65	99.72	99.57	99.72	100	99.57	99.72	99.65	99.5	99.5	99.5	99.65	99.65	99.72	99.65	99.72	99.65	99.72	99.01	98.94
	A/Kelp Gull/Harpon Bay/133943/2023	99.79	99.72	99.57	99.72	99.57	100	99.72	99.65	99.5	99.5	99.5	99.65	99.65	99.72	99.65	99.72	99.65	99.72	99.01	98.94
	A/Brown Skua/Hound Bay/133947/2023	99.79	100	99.86	100	99.72	99.72	100	99.93	99.79	99.79	99.79	99.93	99.93	100	99.93	100	99.93	100	99.29	99.22
	A/Brown Skua/Hound Bay/133949/2023	99.72	99.93	99.93	99.93	99.65	99.65	99.93	100	99.72	99.72	99.72	99.86	99.86	99.93	99.86	99.93	99.86	99.93	99.22	99.15
	A/Brown skua/Bird Island/128289/2023	99.57	99.79	99.65	99.79	99.5	99.5	99.79	99.72	100	99.86	99.86	99.72	99.72	99.79	99.72	99.79	99.72	99.79	99.08	99.01
	A/Brown skua/Bird Island/128288/2023	99.57	99.79	99.65	99.79	99.5	99.5	99.79	99.72	99.86	100	100	99.72	99.72	99.79	99.72	99.79	99.72	99.79	99.08	99.01
	A/Brown skua/Bird Island/128287/2023	99.57	99.79	99.65	99.79	99.5	99.5	99.79	99.72	99.86	100	100	99.72	99.72	99.79	99.72	99.79	99.72	99.79	99.08	99.01
	A/Kelp Gull/Penguin River/141239/2023	99.72	99.93	99.79	99.93	99.65	99.65	99.93	99.86	99.72	99.72	99.72	100	100	99.93	100	99.93	100	99.93	99.22	99.15
	A/Brown Skua/Penguin River/141236/2023	99.72	99.93	99.79	99.93	99.65	99.65	99.93	99.86	99.72	99.72	99.72	100	100	99.93	100	99.93	100	99.93	99.22	99.15
	A/Kelp Gull/Penguin River/141234/2023	99.79	100	99.86	100	99.72	99.72	100	99.93	99.79	99.79	99.79	99.93	99.93	100	99.93	100	99.93	100	99.29	99.22
	A/Brown Skua/Bird Island/141232/2023	99.72	99.93	99.79	99.93	99.65	99.65	99.93	99.86	99.72	99.72	99.72	100	100	99.93	100	99.93	100	99.93	99.22	99.15
	A/Southern Elephant Seal/Jason Harbour/141078/2023	99.79	100	99.86	100	99.72	99.72	100	99.93	99.79	99.79	99.79	99.93	99.93	100	99.93	100	99.93	100	99.29	99.22
	A/Antarctic Fur Seal/Jason Harbour/141037/2023	99.72	99.93	99.79	99.93	99.65	99.65	99.93	99.86	99.72	99.72	99.72	100	100	99.93	100	99.93	100	99.93	99.22	99.15
	A/Southern Elephant Seal/Jason Harbour/141027/2023	99.79	100	99.86	100	99.72	99.72	100	99.93	99.79	99.79	99.79	99.93	99.93	100	99.93	100	99.93	100	99.29	99.22
	A/Southern Fulmar/Falkland Islands/138870/2023	99.08	99.29	99.15	99.29	99.01	99.01	99.29	99.22	99.08	99.08	99.08	99.22	99.22	99.29	99.22	99.29	99.22	99.29	100	99.65
	A/Southern fulmar/Falkland Islands/133789/2023	99.01	99.22	99.08	99.22	98.94	98.94	99.22	99.15	99.01	99.01	99.01	99.15	99.15	99.22	99.15	99.22	99.15	99.22	99.65	100
MP	A/Kelp Gull/Hound Bay/133744/2023	100	99.9	99.8	99.9	99.89	99.9	99.8	99.8	99.9	99.8	99.8	99.9	99.8	99.9	99.8	99.59	99.9	99.8	99.39	99.19
	A/Kelp Gull/Hound Bay/133747/2023	99.9	100	99.9	100	100	100	99.8	99.9	100	99.9	99.9	100	99.9	100	99.9	99.69	100	99.9	99.49	99.29
	A/Brown Skua/Moltke Harbour/133752/2023	99.8	99.9	100	99.9	99.89	99.9	99.69	100	99.9	99.8	99.8	99.9	99.8	99.9	99.8	99.59	99.9	99.8	99.39	99.19
	A/Kelp Gull/Moltke Harbour/133754/2023	99.9	100	99.9	100	100	100	99.8	99.9	100	99.9	99.9	100	99.9	100	99.9	99.69	100	99.9	99.49	99.29
	A/Brown Skua/Moltke Harbour/133755/2023	99.89	100	99.89	100	100	100	99.79	99.89	100	99.89	99.89	100	99.89	100	99.89	99.68	100	99.89	99.47	99.26
	A/Kelp Gull/Harpon Bay/133943/2023	99.9	100	99.9	100	100	100	99.8	99.9	100	99.9	99.9	100	99.9	100	99.9	99.69	100	99.9	99.49	99.29
	A/Brown Skua/Hound Bay/133947/2023	99.8	99.8	99.69	99.8	99.79	99.8	100	99.69	99.8	99.69	99.69	99.8	99.69	99.8	99.69	99.49	99.8	99.69	99.49	99.29
	A/Brown Skua/Hound Bay/133949/2023	99.8	99.9	100	99.9	99.89	99.9	99.69	100	99.9	99.8	99.8	99.9	99.8	99.9	99.8	99.59	99.9	99.8	99.39	99.19
	A/Brown skua/Bird Island/128289/2023	99.9	100	99.9	100	100	100	99.8	99.9	100	99.9	99.9	100	99.9	100	99.9	99.69	100	99.9	99.49	99.29
	A/Brown skua/Bird Island/128288/2023	99.8	99.9	99.8	99.9	99.89	99.9	99.69	99.8	99.9	100	100	99.9	99.8	99.9	99.8	99.59	99.9	99.8	99.39	99.19

	A/Brown skua/Bird Island/128287/2023	99.8	99.9	99.8	99.9	99.89	99.9	99.69	99.8	99.9	100	100	99.9	99.8	99.9	99.8	99.59	99.9	99.8	99.39	99.19
	A/Kelp Gull/Penguin River/141239/2023	99.9	100	99.9	100	100	100	99.8	99.9	100	99.9	99.9	100	99.9	100	99.9	99.69	100	99.9	99.49	99.29
	A/Brown Skua/Penguin River/141236/2023	99.8	99.9	99.8	99.9	99.89	99.9	99.69	99.8	99.9	99.8	99.8	99.9	100	99.9	100	99.59	99.9	99.8	99.39	99.19
	A/Kelp Gull/Penguin River/141234/2023	99.9	100	99.9	100	100	100	99.8	99.9	100	99.9	99.9	100	99.9	100	99.9	99.69	100	99.9	99.49	99.29
	A/Brown Skua/Bird Island/141232/2023	99.8	99.9	99.8	99.9	99.89	99.9	99.69	99.8	99.9	99.8	99.8	99.9	100	99.9	100	99.59	99.9	99.8	99.39	99.19
	A/Southern Elephant Seal/Jason Harbour/141078/2023	99.59	99.69	99.59	99.69	99.68	99.69	99.49	99.59	99.69	99.59	99.59	99.69	99.59	99.69	99.59	100	99.69	99.8	99.19	98.98
	A/Antarctic Fur Seal/Jason Harbour/141037/2023	99.9	100	99.9	100	100	100	99.8	99.9	100	99.9	99.9	100	99.9	100	99.9	99.69	100	99.9	99.49	99.29
	A/Southern Elephant Seal/Jason Harbour/141027/2023	99.8	99.9	99.8	99.9	99.89	99.9	99.69	99.8	99.9	99.8	99.8	99.9	99.8	99.9	99.8	99.8	99.9	100	99.39	99.19
	A/Southern Fulmar/Falkland Islands/138870/2023	99.39	99.49	99.39	99.49	99.47	99.49	99.49	99.39	99.49	99.39	99.39	99.49	99.39	99.49	99.39	99.19	99.49	99.39	100	99.59
	A/Southern fulmar/Falkland Islands/133789/2023	99.19	99.29	99.19	99.29	99.26	99.29	99.29	99.19	99.29	99.19	99.19	99.29	99.19	99.29	99.19	98.98	99.29	99.19	99.59	100
	A/Kelp Gull/Hound Bay/133744/2023	100	100	99.88	99.88	100	100	100	100	99.88	99.88	99.88	100	100	100	100	99.76	100	99.88	99.28	99.28
	A/Kelp Gull/Hound Bay/133747/2023	100	100	99.88	99.88	100	100	100	100	99.88	99.88	99.88	100	100	100	100	99.76	100	99.88	99.28	99.28
	A/Brown Skua/Moltke Harbour/133752/2023	99.88	99.88	100	99.76	99.88	99.88	99.88	99.88	99.76	99.76	99.76	99.88	99.88	99.88	99.88	99.64	99.88	99.76	99.16	99.16
	A/Kelp Gull/Moltke Harbour/133754/2023	99.88	99.88	99.76	100	99.88	99.88	99.88	99.88	99.76	99.76	99.76	99.88	99.88	99.88	99.88	99.64	99.88	99.76	99.16	99.16
	A/Brown Skua/Moltke Harbour/133755/2023	100	100	99.88	99.88	100	100	100	100	99.88	99.88	99.88	100	100	100	100	99.76	100	99.88	99.28	99.28
	A/Kelp Gull/Harpon Bay/133943/2023	100	100	99.88	99.88	100	100	100	100	99.88	99.88	99.88	100	100	100	100	99.76	100	99.88	99.28	99.28
	A/Brown Skua/Hound Bay/133947/2023	100	100	99.88	99.88	100	100	100	100	99.88	99.88	99.88	100	100	100	100	99.76	100	99.88	99.28	99.28
	A/Brown Skua/Hound Bay/133949/2023	100	100	99.88	99.88	100	100	100	100	99.88	99.88	99.88	100	100	100	100	99.76	100	99.88	99.28	99.28
	A/Brown skua/Bird Island/128289/2023	99.88	99.88	99.76	99.76	99.88	99.88	99.88	99.88	100	100	100	99.88	99.88	99.88	99.88	99.64	99.88	99.76	99.16	99.16
NS	A/Brown skua/Bird Island/128288/2023	99.88	99.88	99.76	99.76	99.88	99.88	99.88	99.88	100	100	100	99.88	99.88	99.88	99.88	99.64	99.88	99.76	99.16	99.16
	A/Brown skua/Bird Island/128287/2023	99.88	99.88	99.76	99.76	99.88	99.88	99.88	99.88	100	100	100	99.88	99.88	99.88	99.88	99.64	99.88	99.76	99.16	99.16
	A/Kelp Gull/Penguin River/141239/2023	100	100	99.88	99.88	100	100	100	100	99.88	99.88	99.88	100	100	100	100	99.76	100	99.88	99.28	99.28
	A/Brown Skua/Penguin River/141236/2023	100	100	99.88	99.88	100	100	100	100	99.88	99.88	99.88	100	100	100	100	99.76	100	99.88	99.28	99.28
	A/Kelp Gull/Penguin River/141234/2023	100	100	99.88	99.88	100	100	100	100	99.88	99.88	99.88	100	100	100	100	99.76	100	99.88	99.28	99.28
	A/Brown Skua/Bird Island/141232/2023	100	100	99.88	99.88	100	100	100	100	99.88	99.88	99.88	100	100	100	100	99.76	100	99.88	99.28	99.28
	A/Southern Elephant Seal/Jason Harbour/141078/2023	99.76	99.76	99.64	99.64	99.76	99.76	99.76	99.76	99.64	99.64	99.64	99.76	99.76	99.76	99.76	100	99.76	99.88	99.05	99.05
	A/Antarctic Fur Seal/Jason Harbour/141037/2023	100	100	99.88	99.88	100	100	100	100	99.88	99.88	99.88	100	100	100	100	99.76	100	99.88	99.28	99.28
	A/Southern Elephant Seal/Jason Harbour/141027/2023	99.88	99.88	99.76	99.76	99.88	99.88	99.88	99.88	99.76	99.76	99.76	99.88	99.88	99.88	99.88	99.88	99.88	100	99.16	99.16
	A/Southern Fulmar/Falkland Islands/138870/2023	99.28	99.28	99.16	99.16	99.28	99.28	99.28	99.28	99.16	99.16	99.16	99.28	99.28	99.28	99.28	99.05	99.28	99.16	100	99.76
	A/Southern fulmar/Falkland Islands/133789/2023	99.28	99.28	99.16	99.16	99.28	99.28	99.28	99.28	99.16	99.16	99.16	99.28	99.28	99.28	99.28	99.05	99.28	99.16	99.76	100

Supplementary Table 4: Amino acid polymorphisms in PB2 at positions associated with mammalian adaptation (271, 591, 627 and 701) in H5N1 HPAIV sequences derived from mammalian samples collected in South Georgia.

Sequence name	Amino acid mammalian adaptation polymorphism in PB2			
	**T271A**	**Q591K**	**E627K**	**D701N**
A/Southern Elephant Seal/Jason Harbour/141078/2023	T	Q	K	D
A/Antarctic Fur Seal/Jason Harbour/141037/2023	T	Q	E	D
A/Southern Elephant Seal/Jason Harbour/141027/2023	T	Q	E	D

Supplementary Table 5: Coverage statistics from whole genome sequencing (WGS) of influenza virus. Quality assessment of the WGS sequence obtained. For each segment the total reads, genome coverage and mean depth of reads were obtained using samtools.

Sample	PB2			PB1			PA			HA			NP			NA			MP			NS		
	Reads	Coverage (%)	Mean depth	Reads	Coverage (%)	Mean depth	Reads	Coverage (%)	Mean depth	Reads	Coverage (%)	Mean depth	Reads	Coverage (%)	Mean depth	Reads	Coverage (%)	Mean depth	Reads	Coverage (%)	Mean depth	Reads	Coverage (%)	Mean depth
A/Brown_skua/Bird_Island/128287/2023	15445	100	6940.48	67276	100	14014.2	7641	100	6720.09	53630	100	20189.5	26360	100	10641.5	43548	100	20878.1	88013	100	82366	154917	100	150789
A/Brown_skua/Bird_Island/128288/2023	10018	100	2301.93	39926	100	8214.75	2635	100	1627.41	53823	100	18871.4	6540	100	2796.93	33318	100	12912.3	41378	100	35496	91534	100	89094
A/Brown_skua/Bird_Island/128289/2023	150548	100	26613.2	183077	100	33444.1	62339	100	21845.3	81854	100	31548	5975	100	5528.35	88258	100	42637.4	156285	100	145460	291252	100	275522
A/Southern_fulmar/Falkland_Islands/133789/2023	7117	100	2521.37	7490	100	3785.91	6875	100	4274.6	42297	100	14267.5	9874	100	7606.65	20983	100	9429.58	34693	100	28196	48784	100	43657
A/Southern_Elephant_Seal/Jason_Harbour/141078/2023	77854	100	8280.18	131085	100	12614.2	84715	100	10383.1	3299	100	980.214	1270	100	1083.03	2573	100	1217.09	7522	100	6573.8	10534	100	9551.4
A/Antarctic_Fur_Seal/Jason_Harbour/141037/2023	5209	100	1092.46	14622	100	2432.99	5238	100	1184.95	6760	100	1794.37	1523	100	769.025	4700	100	1844.01	7282	100	5078.3	12247	100	9097.9
A/Southern_Elephant_Seal/Jason_Harbour/141027/2023	6862	100	720.391	6883	100	921.895	5264	100	886.007	2049	98.93	555.993	932	100	796.944	952	100	393.292	2310	100	1852.8	4193	100	3687.5
A/Brown_Skua/Penguin_River/141236/2023	1627	100	329.997	3601	99.872	616.627	1295	100	317.77	1519	100	404.896	422	100	185.876	1105	100	428.149	1773	100	1218.8	2873	100	2152.4
A/Kelp_Gull/Penguin_River/141234/2023	1451	100	386.294	4439	100	857.819	1677	100	533.127	2549	100	746.119	678	100	443.424	1789	100	753.149	4335	100	3576	6325	100	5422.6
A/Brown_Skua/Bird_Island/141232/2023	2572	100	517.941	6559	100	1112.38	2371	100	589.732	3673	100	818.708	629	100	333.523	1935	100	733.156	3449	100	2331.8	6191	100	4090
A/Kelp_Gull/Penguin_River/141239/2023	1950	100	452.795	6108	100	1004.29	2182	100	552.227	2374	100	694.721	638	100	403.801	2419	100	973.629	3385	100	2409.2	5179	100	4001.9
A/Brown_Skua/Penguin_River/141240/2023	38441	100	10147.8	93973	100	23102.5	25487	100	9179.06	155400	100	51274.9	36935	100	26682.1	109978	100	46529.5	185374	100	165804	422546	100	397071
A/South_Georgia_Shag/King_Edward_Cove/141245/2023	110921	100	26666.2	454414	100	75602.1	151232	100	37659.4	152245	100	42438.5	24862	100	14613.3	142366	100	57916.6	194922	100	145701	271286	100	232400
A/Southern_Fulmar/Falkland_Islands/138870/2023	612101	100	32568.3	516329	100	28255.4	552035	100	30910.1	503783	100	34003.3	443806	100	35411.6	365059	100	30656.5	156767	100	18724	87122	100	12030
A/Kelp_Gull/Hound_Bay/133744/2023	2570	99.701	135.801	2371	99.317	126.204	1806	99.821	101.549	2677	99.775	184.956	2232	97.189	182.154	1590	99.52	128.805	916	100	111.79	549	98.194	76.917
A/Kelp_Gull/Hound_Bay/133747/2023	815	95.088	43.5681	659	94.02	34.7945	653	99.418	36.3511	859	99.775	60.2427	646	97.189	53.6613	406	97.737	33.9986	243	99.026	29.294	132	100	17.525
A/Brown_Skua/Moltke_Harbour/133752/2023	1011	99.317	53.9624	749	96.027	39.2952	901	99.687	49.2141	1351	99.606	92.3716	1110	99.361	89.7808	731	99.589	59.631	394	100	48.304	220	98.262	32.634
A/Kelp_Gull/Moltke_Harbour/133754/2023	1197	98.847	67.6181	861	96.241	47.0662	917	99.239	54.0506	1288	99.606	94.1256	1113	94.633	93.5227	787	99.931	67.7215	376	98.539	47.725	253	98.646	35.921
A/Brown_Skua/Moltke_Harbour/133755/2023	1631	98.719	93.9671	1543	97.693	88.8065	1156	99.731	68.6955	1526	99.775	114.944	1494	98.083	128.494	683	99.931	61.6962	374	95.91	47.82	268	100	39.376

A/Brown_Skua/Hound_Bay/133947/2023	374151	100	20873	299020	100	16817.8	327445	100	19232.6	303779	100	22155.8	268166	100	21939.4	227658	100	19756.7	79674	100	9983.6	41730	100	6271.3
A/Brown_Skua/Hound_Bay/133949/2023	290600	100	16436.4	221570	99.829	12657.3	239059	100	14153.4	245924	100	18119	218527	100	18236.5	180110	100	15861.5	69124	100	8800	33789	100	5093.5
A/Kelp_Gull/Harpon_Bay/133943/2023	338135	100	18925.8	271653	100	15349.1	257778	100	15147.8	259086	100	19009.9	236530	100	19507.7	190288	100	16636.7	65064	100	8099.6	32739	100	4878
A/Antarctic_tern/King_Edward_Point/141271/2023	91595	100	4954.25	66717	100	3644.45	78631	100	4543.74	73409	99.775	5157.89	61979	100	5042.22	48879	100	4169.25	18316	100	2262.1	12739	100	1728.8

Supplementary Table 6: Oligonucleotides used in this study. FAM, Fluorescein; BHQ-1, black hole quencher 1; NFQ-MGB, nonfluorescent quencher-minor groove binder; APMV-1, avian paramyxovirus type-1.

Purpose	ID	Type	Sequence and modification (5'-3')	Author	Refence
M-gene RRT-PCR	SVIP-MP-F	Primer	GGC CCC CTC AAA GCC GA	Nagy et al.	https://doi.org/10.1371/journal.pone.0244669
	SVIP-MP-R	Primer	CGT CTA CGY TGC AGT CC		
	SVIP-MP-P2-MGB	Probe	FAM-TCA CTK GGC ACG GTG AGC GT-NFQ-MGB		
	Sep1	Primer	AGA TGA GTC TTC TAA CCG AGG TCG	Spackman et al.	https://doi.org/10.1128/jcm.40.9.3256-3260.2002
	Sep2	Primer	TGC AAA AAC ATC TTC AAG TCT CTG		
	SePRO	Probe	FAM-TCA GGC CCC CTC AAA GCC GA-TAMRA		
H5-HP RRT-PCR	2344b_H5_F3	Primer	CCT TGC GAC TGG GCT CAG	James et al.	https://doi.org/10.1016/j.jviromet.2022.114454
	2344b_H5_R2	Primer	ATC AAC CAT TCC CTG CCA		
	2344b_DE_Sonde	Probe	FAM-AGA AGA AAR AGA GGG CTG TTT GGG GCT-BHQ-1		
N1 RRT-PCR	N1F2	Primer	GTT TGA GTC TGT TGC TTG GTC	Payungporn et al.	https://doi.org/10.1016/j.jviromet.2005.08.004
	N1R1	Primer	TGA TAG TGT CTG TTA TTA TGC C		
	N1 probe	Probe	VIC-TTG TAT TTC AAT ACA GCC AC-NFQ-MGB		
APMV-1 RRT-PCR	NDF	Primer	GAG CTA ATG AAC ATT CTT TC	Sutton et al.	https://doi.org/10.1016/j.jviromet.2018.12.001
	NDR	Primer	AAT AGG CGG ACC ACA TC TG		
	LproMGB	Probe	FAM-CCA ATC AAC TTC CC-NFQ-MGB		
	LproMGB2	Probe	VIC-AAT AGT GTA TGA CAA CAC-NFQ-MGB		
WGS cDNA synthesis	Optil-F1	Primer	TTACGCGCCAGCAAAAGCAG	Chrzastek et al.<br>Leyson et al.	https://doi.org/10.1016/j.virol.2017.06.019<br>https://doi.org/10.1016/j.virol.2019.08.020
	Optil-F2	Primer	GTTACGCGCCAGCGAAAGCAGG		
	Optil-R1	Primer	GTTACGCGCCAGTAGAAACAAG		

Supplementary Table 7: GISAID reference numbers and isolate names for all sequences used to align to sequences generated during this study.

Isolate_Id	Isolate_Name
EPI_ISL_10220427	A/red_fox/Netherlands/22002636-005/2022_|H5N1|_2022-02-07
EPI_ISL_10993074	A/Mallard/Netherlands/2/2022_|H5N1|_2022-02-14
EPI_ISL_11007539	A/turkey/Bulgaria/755-1_22VIR778-4/2021_|H5N1|_2021-11-30
EPI_ISL_11007542	A/chicken/Niger/22VIR1409-13/2022_|H5N1|_2022-01-01
EPI_ISL_11007547	A/chicken/Niger/22VIR1409-9/2022_|H5N1|_2022-01-01
EPI_ISL_11112342	A/Barnacle_Goose/Netherlands/11/2022_|H5N1|_2022-02-09
EPI_ISL_11112358	A/European_Herring_Gull/Netherlands/2/2022_|H5N1|_2022-02-26
EPI_ISL_11211731	A/duck/Bangladesh/19D1818/2021_|H5N1|_2021-12-22
EPI_ISL_11259275	A/whooper_swan/Ireland/032444_22VIR1325-2/2021_|H5N1|_2021-11-09
EPI_ISL_11259299	A/turkey/Spain/490-22_22VIR2142-26/2022_|H5N1|_2022-02-08
EPI_ISL_11259304	A/chicken/Spain/564-4_22VIR2142-30/2022_|H5N1|_2022-02-11
EPI_ISL_11259315	A/goose/Spain/88-3_22VIR2142-4/2022_|H5N1|_2022-01-10
EPI_ISL_11327088	A/chicken/Czech_Republic/61-1/2022_|H5N1|_2021-12-31
EPI_ISL_11330431	A/white-tailed_eagle/Hokkaido/22-RU-WTE-2/2022_|H5N1|_2022-01-02
EPI_ISL_11561593	A/black-headed_gull/England/388256/2022_|H5N1|_2022-02-03
EPI_ISL_11633709	A/Cygnus_columbianus/Hubei/128/2021_|H5N1|_2021-12-20
EPI_ISL_11798571	A/common_buzzard/Denmark/24271-1.02/2021-10-25_|H5N1|_2021-10-25
EPI_ISL_11798572	A/Eurasian_wigeon/Denmark/24066-9/2021-10-13_|H5N1|_2021-10-13
EPI_ISL_11922811	A/goose/Poland/H124_22VIR2515-5/2022_|H5N1|_2022-02-05
EPI_ISL_11922815	A/swan/Romania/16905_22VIR2749-1/2021_|H5N1|_2021-12-08
EPI_ISL_11922817	A/swan/Romania/10394_22VIR2749-3/2022_|H5N1|_2022-02-03
EPI_ISL_11971481	A/turkey/South_Dakota/22-008485-001-original/2022_|H5N1|_2022-03-21
EPI_ISL_12069289	A/Fox/Netherlands/EMC3/2022_|H5N1|_2022-01-22
EPI_ISL_12174842	A/Ezo_red_fox/Hokkaido/1/2022_|H5N1|_2022-03-31
EPI_ISL_12223734	A/grey_heron/Czech_Republic/25338-2/2021_|H5N1|_2021-12-18
EPI_ISL_12514573	A/Song_Thrush/Netherlands/1/2022_|H5N1|_2022-04-13

EPI_ISL_12572655	A/duck/Guangdong/S4525/2021_|H5N1|_2021-12-08
EPI_ISL_12572659	A/duck/Hubei/SE220/2022_|H5N1|_2022-01-10
EPI_ISL_12572661	A/goose/Guizhou/S1541/2022_|H5N1|_2022-02-22
EPI_ISL_12572662	A/goose/Hunan/SE284/2022_|H5N1|_2022-01-05
EPI_ISL_12690905	A/chicken/New_York/22-009324-002-original/2022_|H5N1|_2022-03-28
EPI_ISL_12749687	A/turkey/Israel/537/2021_|H5N1|_2021-10-12
EPI_ISL_12917757	A/turkey/Israel/564/2021_|H5N1|_2021-10-29
EPI_ISL_12968592	A/Fancy_chicken/NL/FAV-0035/2021_|H5N1|_2021-12-17
EPI_ISL_13026106	A/Canada_goose/Luxembourg/21239614/2021_|H5N1|_2021-12-10
EPI_ISL_13052412	A/turkey/Maine/22-009605-001-original/2022_|H5N1|_2022-03-28
EPI_ISL_13052728	A/fox/Wisconsin/22-014746-029-original/2022_|H5N1|_2022-05-06
EPI_ISL_13199866	A/sandwich_tern/Netherlands/22010161-001005/2022_|H5N1|_2022-05-30
EPI_ISL_13285238	A/turkey/South_Dakota/22-009980-002-original/2022_|H5N1|_2022-04-01
EPI_ISL_13285245	A/pheasant/Texas/22-009984-001-original/2022_|H5N1|_2022-03-29
EPI_ISL_13352721	A/whooper_swan/Henan/45/2021_|H5N1|_2021-12-17
EPI_ISL_13352728	A/mandarin_duck/Henan/426/2021_|H5N1|_2021-12-17
EPI_ISL_13369746	A/chicken/England/057016/2021_|H5N1|_2021-11-12
EPI_ISL_13369753	A/barnacle_goose/Scotland/292583/2021_|H5N1|_2021-11-12
EPI_ISL_13369780	A/barnacle_goose/England/293809/2021_|H5N1|_2021-11-26
EPI_ISL_13370519	A/domestic_duck/England/066456/2021_|H5N1|_2021-12-06
EPI_ISL_13370564	A/chicken/Northern_Ireland/17903/2021_|H5N1|_2021-12-09
EPI_ISL_13370573	A/chicken/England/070277/2021_|H5N1|_2021-12-13
EPI_ISL_13370696	A/turkey/England/007586/2022_|H5N1|_2022-01-23
EPI_ISL_13370914	A/domestic_duck/England/029617/2022_|H5N1|_2022-03-20
EPI_ISL_13370918	A/domestic_duck/England/041295/2022_|H5N1|_2022-04-04
EPI_ISL_13429293	A/Black-headed_gull/Netherlands/5/2022_|H5N1|_2022-06-03
EPI_ISL_13432604	A/turkey/South_Dakota/22-010139-002-original/2022_|H5N1|_2022-04-04
EPI_ISL_13453568	A/turkey/England/081490/2022_|H5N1|_2022-06-05
EPI_ISL_13486831	A/Common_eider/England/023549/2022_|H5N1|_2022-03-03
EPI_ISL_13778474	A/European_Herring_Gull/Netherlands/7/2022_|H5N1|_2022-06-07

EPI_ISL_13955204	A/chicken/Czech_Republic/8028-1/2022_|H5N1|_2022-04-13
EPI_ISL_13969426	A/Gannet/Scotland/090910/2022_|H5N1|_2022-06-18
EPI_ISL_13990715	A/Cygnus_olor/Spain/1675-1_22VIR6312-14/2022_|H5N1|_2022-04-26
EPI_ISL_13990718	A/Gypaetus_barbatus/Spain/1956-25_22VIR6312-19/2022_|H5N1|_2022-05-27
EPI_ISL_14064890	A/crow/Hokkaido/0103B073/2022_|H5N1|_2022-04-01
EPI_ISL_14065167	A/white-tailed_eagle/Hokkaido/20220210001/2022_|H5N1|_2022-02-10
EPI_ISL_14070455	A/tanuki/Hokkaido/1/2022_|H5N1|_2022-04-01
EPI_ISL_14233925	A/Common_Tern/Netherlands/20/2022_|H5N1|_2022-07-22
EPI_ISL_14393465	A/Branta_canadensis/Belgium/4821_0001/2022_|H5N1|_2022-04-07
EPI_ISL_14497855	A/domestic_duck/England/104859/2022_|H5N1|_2022-08-05
EPI_ISL_14542488	A/chicken/South_Africa/26683/2021_|H5N1|_2021-04-09
EPI_ISL_14571430	A/common_tern/Germany-NI/AI03185/2022_|H5N1|_2022-06-07
EPI_ISL_14573124	A/Egyptian_goose/South_Africa/21050245A/2021_|H5N1|_2021-05-13
EPI_ISL_14619009	A/Hartlaubs_gull/South_Africa/21050295/2021_|H5N1|_2021-05-17
EPI_ISL_14619020	A/chicken/South_Africa/21050293/2021_|H5N1|_2021-05-17
EPI_ISL_14619024	A/chicken/South_Africa/21050299/2021_|H5N1|_2021-05-18
EPI_ISL_14620233	A/Buff_Orpington_chicken/South_Africa/21050364/2021_|H5N1|_2021-05-19
EPI_ISL_14638201	A/Hartlaubs_gull/South_Africa/21050388/2021_|H5N1|_2021-05-20
EPI_ISL_14638909	A/African_penguin/South_Africa/21050383/2021_|H5N1|_2021-05-20
EPI_ISL_14639383	A/Kelp_gull/South_Africa/21050387/2021_|H5N1|_2021-05-20
EPI_ISL_14639837	A/Spotted_eagle_owl/South_Africa/21050363/2021_|H5N1|_2021-05-20
EPI_ISL_14644592	A/African_barn_owl/South_Africa/21050429/2021_|H5N1|_2021-05-25
EPI_ISL_14645331	A/Black-headed_heron/South_Africa/2021_|H5N1|_2021-05-25
EPI_ISL_14645717	A/chicken/South_Africa/693781/2021_|H5N1|_2021-05-27
EPI_ISL_14646080	A/pelican/South_Africa/21050494/2021_|H5N1|_2021-05-29
EPI_ISL_14710708	A/duck/South_Africa/21060064/2021_|H5N1|_2021-06-03
EPI_ISL_14710814	A/African_fish_eagle/South_Africa/21060065/2021_|H5N1|_2021-06-03
EPI_ISL_14760611	A/goose/Italy/IZSLT_21VIR10273/2021_|H5N1|_2021-11-22
EPI_ISL_14760625	A/chicken/Italy/21VIR10096/2021_|H5N1|_2021-11-23
EPI_ISL_14760649	A/turkey/Italy/21VIR10396/2021_|H5N1|_2021-11-29

EPI_ISL_14760654	A/chicken/Italy/21VIR10470/2021_|H5N1|_2021-11-30
EPI_ISL_14761097	A/turkey/Italy/21VIR11587-1/2021_|H5N1|_2021-12-23
EPI_ISL_14761272	A/turkey/Italy/IZSLT_22VIR366-3/2022_|H5N1|_2022-01-04
EPI_ISL_14761373	A/buzzard/Italy/22VIR767-2/2022_|H5N1|_2022-01-24
EPI_ISL_14761407	A/duck/Italy/22VIR1294-1/2022_|H5N1|_2022-02-08
EPI_ISL_14761428	A/goose/Italy/22VIR1520/2022_|H5N1|_2022-02-14
EPI_ISL_14761496	A/chicken/Italy/22VIR1953-4/2022_|H5N1|_2022-02-22
EPI_ISL_14769967	A/chicken/Lesotho/352.3/2021_|H5N1|_2021-06-05
EPI_ISL_14871335	A/poultry/Benin/21-A-08-035-O/2021_|H5N1|_2021-09-01
EPI_ISL_14917915	A/breeding_hen/Poland/H361/2022_|H5N1|_2022-07-18
EPI_ISL_14918031	A/Sacred_ibis/South_Africa/21060395/2021_|H5N1|_2021-06-19
EPI_ISL_14918305	A/ostrich/South_Africa/21060357/2021_|H5N1|_2021-06-21
EPI_ISL_14918893	A/Blue_crane/South_Africa/21070050/2021_|H5N1|_2021-07-01
EPI_ISL_14918903	A/chicken/South_Africa/411255/2021_|H5N1|_2021-07-19
EPI_ISL_14933722	A/ostrich/South_Africa/21070586/2021_|H5N1|_2021-07-28
EPI_ISL_14933723	A/chicken/South_Africa/412372/2021_|H5N1|_2021-07-28
EPI_ISL_14934607	A/chicken/South_Africa/MAB/2021_|H5N1|_2021-08-27
EPI_ISL_14934884	A/chicken/South_Africa/412381/2021_|H5N1|_2021-09-08
EPI_ISL_14973559	A/Cape_gannet/South_Africa/21100108/2021_|H5N1|_2021-10-07
EPI_ISL_14973864	A/Cape_cormorant/South_Africa/21100109A/2021_|H5N1|_2021-10-07
EPI_ISL_15004397	A/turkey/Kansas/22-011119-004-original/2022_|H5N1|_2022-04-09
EPI_ISL_15038822	A/chicken/England/114595/2022_|H5N1|_2022-09-02
EPI_ISL_15063845	A/snow_goose/Kansas/W22-177B/2022_|H5N1|_2022-03-04
EPI_ISL_15072538	A/chicken/Hokkaido/I-1/2022_|H5N1|_2022-04-16
EPI_ISL_15072539	A/emu/Hokkaido/A-2/2022_|H5N1|_2022-04-16
EPI_ISL_15072540	A/crow/Hokkaido/HU-1/2022_|H5N1|_2022-05-18
EPI_ISL_15078246	A/fox/Wisconsin/22-014746-008-original/2022_|H5N1|_2022-05-03
EPI_ISL_15088302	A/Greylag_Goose/Netherlands/13/2022_|H5N1|_2022-08-27
EPI_ISL_15088303	A/Lesser_Black-backed_Gull/Netherlands/3/2022_|H5N1|_2022-08-29
EPI_ISL_15267014	A/Eurasian_Spoonbill/Netherlands/6/2022_|H5N1|_2022-06-16

EPI_ISL_15364797	A/Mute_Swan/Netherlands/2/2022_|H5N1|_2022-09-19
EPI_ISL_15518267	A/ural_owl/Finland/14041_21RS3535-7/2021_|H5N1|_2021-10-01
EPI_ISL_15535272	A/domestic_goose/Poland/H397-N/2022_|H5N1|_2022-09-20
EPI_ISL_15576616	A/white-fronted_goose/Miyagi/0410D001/2022_|H5N1|_2022-10-04
EPI_ISL_15581789	A/Mallard/Netherlands/19/2022_|H5N1|_2022-10-12
EPI_ISL_15585755	A/Chicken/England/129801/2022_|H5N1|_2022-10-10
EPI_ISL_15585763	A/Turkey/England/127741/2022_|H5N1|_2022-10-06
EPI_ISL_15585843	A/Turkey/England/130119/2022_|H5N1|_2022-10-10
EPI_ISL_15585883	A/chicken/England/131258/2022_|H5N1|_2022-10-13
EPI_ISL_15586107	A/Bufflehead_Duck/Northern_Ireland/012321/2022_|H5N1|_2022-10-10
EPI_ISL_15586114	A/Domestic_Duck/England/134267/2022_|H5N1|_2022-10-18
EPI_ISL_15614803	A/ibis/Egypt/RLQP-229S/2022_|H5N1|_2022-06-17
EPI_ISL_15647836	A/Mandarin_duck/Korea/WA496/2022_|H5N1|_2022-10-10
EPI_ISL_15676638	A/mute_swan/Croatia/108/2022_|H5N1|_2022-10-18
EPI_ISL_15812579	A/Anser_anser_domesticus/Belgium/10918_0002/2022_|H5N1|_2022-09-16
EPI_ISL_15813940	A/mixed_domestic_Anatidae/Belgium/12318_0001/2022_|H5N1|_2022-10-24
EPI_ISL_15814043	A/Gallus_gallus/Belgium/12395_0002/2022_|H5N1|_2022-10-24
EPI_ISL_15837655	A/chicken/South_Africa/21080238/2021_|H5N1|_2022-10-10
EPI_ISL_15837851	A/Cape_cormorant/South_Africa/21100176B/2021_|H5N1|_2021-10-11
EPI_ISL_15838197	A/White_breasted_cormorant/South_Africa/21100215/2021_|H5N1|_2021-10-13
EPI_ISL_15838583	A/Northern_giant_petrel/South_Africa/21100283/2021_|H5N1|_2021-10-16
EPI_ISL_15838771	A/chicken/South_Africa/236744/2021_|H5N1|_2021-10-19
EPI_ISL_15852340	A/cormorant/South_Africa/21120147/2021_|H5N1|_2021-12-06
EPI_ISL_15852552	A/Kelp_gull/South_Africa/22010082/2022_|H5N1|_2022-01-07
EPI_ISL_15852846	A/chicken/South_Africa/QF22465/2022_|H5N1|_2022-02-07
EPI_ISL_15853343	A/wild_duck/South_Africa/UP196/2022_|H5N1|_2022-02-15
EPI_ISL_15853608	A/Common_tern/South_Africa/681998_CT010/2022_|H5N1|_2022-03-21
EPI_ISL_15853610	A/Swift_tern/South_Africa/681998_ST009/2022_|H5N1|_2022-03-22
EPI_ISL_15853701	A/Cape_cormorant/South_Africa/681998_DOA059/2022_|H5N1|_2022-03-23
EPI_ISL_15853835	A/African_black_oystercatcher/South_Africa/682489/2022_|H5N1|_2022-03-28

EPI_ISL_15853920	A/Common_tern/South_Africa/686940_CT013/2022_|H5N1|_2022-05-10
EPI_ISL_15857846	A/turkey/Italy/22VIR10627-4/2022_|H5N1|_2022-11-02
EPI_ISL_15857961	A/Goose/Italy/22VIR9673-3/2022_|H5N1|_2022-10-09
EPI_ISL_15857991	A/Hawaiian_goose/Italy/22VIR9126-7/2022_|H5N1|_2022-09-23
EPI_ISL_15925881	A/Gadwall/Netherlands/2/2022_|H5N1|_2022-11-09
EPI_ISL_15925882	A/Eurasian_Wigeon/Netherlands/3/2022_|H5N1|_2022-10-21
EPI_ISL_15925885	A/Black-headed_Gull/Netherlands/11/2022_|H5N1|_2022-11-11
EPI_ISL_15943015	A/Wild_bird/Korea/K22-742/2022_|H5N1|_2022-11-07
EPI_ISL_15944663	A/Spot-billed_duck/Korea/K22-856-2/2022_|H5N1|_2022-11-17
EPI_ISL_15944667	A/Spot-billed_duck/Korea/K22-920/2022_|H5N1|_2022-11-21
EPI_ISL_15957744	A/Chicken/Scotland/141941/2022_|H5N1|_2022-10-30
EPI_ISL_15957798	A/Turkey/England/143029/2022_|H5N1|_2022-11-01
EPI_ISL_15957899	A/turkey/England/146512/2022_|H5N1|_2022-11-06
EPI_ISL_16013752	A/Pelican/Venezuela/Pel3/2022_|H5N1|_2022-11-25
EPI_ISL_16113231	A/tawny_owl/Denmark/05372-1.01/2022_|H5N1|_2022-06-08
EPI_ISL_16115054	A/common_buzzard/Denmark/08287-1.01/2022_|H5N1|_2022-08-30
EPI_ISL_16171323	A/turkey/Minnesota/22-012126-003-original/2022_|H5N1|_2022-04-19
EPI_ISL_16171452	A/domestic_goose/Michigan/22-012855-004-original/2022_|H5N1|_2022-04-25
EPI_ISL_16183504	A/Chicken/AB/FAV-0222-01/2022_|H5N1|_2022-05-16
EPI_ISL_16196676	A/duck/Cyprus/750-2_22VIR12133-10/2022_|H5N1|_2022-11-25
EPI_ISL_16240382	A/duck/Washington/22-014327-002-original/2022_|H5N1|_2022-05-07
EPI_ISL_16243273	A/fox/New_York/22-015065-001-original/2022_|H5N1|_2022-04-27
EPI_ISL_16243894	A/chicken/Idaho/22-015271-002-original/2022_|H5N1|_2022-05-16
EPI_ISL_16249681	A/chicken/Peru/LAM-INS-002/2022_|H5N1|_2022-11-28
EPI_ISL_16297063	A/chicken/Montana/22-034813-003-original/2022_|H5N1|_2022-10-27
EPI_ISL_16297332	A/goose/Louisiana/22-033391-001-original/2022_|H5N1|_2022-10-14
EPI_ISL_16384139	A/domestic_goose/England/121576/2022_|H5N1|_2022-09-22
EPI_ISL_16384145	A/Chicken/Scotland/153380/2022_|H5N1|_2022-11-19
EPI_ISL_16384147	A/Chicken/England/155942/2022_|H5N1|_2022-11-20
EPI_ISL_16384178	A/canada_goose/England/119770/2022_|H5N1|_2022-11-30

EPI_ISL_16384181	A/mute_swan/England/119920/2022_|H5N1|_2022-12-06
EPI_ISL_16384193	A/mute_swan/England/395769/2022_|H5N1|_2022-11-25
EPI_ISL_16388458	A/whooper_swan/Akita/0511B002/2022_|H5N1|_2022-11-23
EPI_ISL_16507106	A/Ciconia_ciconia/Spain/22VIR12774-30_3394-2/2022_|H5N1|_2022-09-19
EPI_ISL_16524203	A/Black-headed_gull/Netherlands/12/2022_|H5N1|_2022-12-23
EPI_ISL_16524248	A/European_herring_gull/Netherlands/21/2022_|H5N1|_2022-12-27
EPI_ISL_16549349	A/Phasianus_colchicus/Belgium/10993-0003/2022_|H5N1|_2022-11-15
EPI_ISL_16574657	A/greylag_goose/England/120308/2022_|H5N1|_2022-12-15
EPI_ISL_16574736	A/goose/England/396398/2022_|H5N1|_2022-12-17
EPI_ISL_16641777	A/sanderling/MA/22HP00192/2022_|H5N1|_2022-03-03
EPI_ISL_16641785	A/common_tern/ME/COTE_20220614_1/2022_|H5N1|_2022-06-14
EPI_ISL_16641791	A/peregrine_falcon/MA/22MM00756/2022_|H5N1|_2022-06-16
EPI_ISL_16641799	A/northern_gannet/MA/22WI00050/2022_|H5N1|_2022-05-10
EPI_ISL_16676447	A/Common_tern/South_Africa/22060305/2022_|H5N1|_2022-06-17
EPI_ISL_16676471	A/Gallus_gallus/Belgium/00322-0002/2023_|H5N1|_2023-01-10
EPI_ISL_16676474	A/Gallus_gallus/Belgium/14733-0001/2022_|H5N1|_2022-12-20
EPI_ISL_16698546	A/eastern_buzzard/Niigata/1501B001/2022_|H5N1|_2022-10-21
EPI_ISL_16741201	A/duck/France/22P025078/2022_|H5N1|_2022-12-06
EPI_ISL_16811193	A/turkey/England/006957/2023_|H5N1|_2023-01-11
EPI_ISL_16838579	A/pelican/Russia/1032-1/2021_|H5N1|_2021-07-16
EPI_ISL_16839065	A/Cormorant/Namibia/141/2022_|H5N1|_2022-01-27
EPI_ISL_16891400	A/gray_gull/Chile/C61947/2022_|H5N1|_2022-12-02
EPI_ISL_16891402	A/black_skimmer/Chile/C61962/2022_|H5N1|_2022-12-02
EPI_ISL_16904552	A/Chicken/Scotland/172652/2022_|H5N1|_2022-12-14
EPI_ISL_16937292	A/chicken/Czech_Republic/179/2023_|H5N1|_2023-01-04
EPI_ISL_16937301	A/chicken/Czech_Republic/22969-5/2022_|H5N1|_2022-12-10
EPI_ISL_16937302	A/chicken/Czech_Republic/22968-3/2022_|H5N1|_2022-12-09
EPI_ISL_16937309	A/environment/Czech_Republic/1220-3_hall-8/2023_|H5N1|_2023-01-23
EPI_ISL_16955748	A/large-billed_crow/Hokkaido/0111E084/2022_|H5N1|_2022-11-24
EPI_ISL_16955765	A/large-billed_crow/Hokkaido/0112P083-2/2022_|H5N1|_2022-12-05

EPI_ISL_16955798	A/slaty-backed_gull/Hokkaido/0111M114/2022_|H5N1|_2022-11-16
EPI_ISL_16979817	A/black-headed_gull/Italy/23VIR1107-4/2022_|H5N1|_2022-12-30
EPI_ISL_16997756	A/chicken/Czech_Republic/1600-1/2023_|H5N1|_2023-01-29
EPI_ISL_17011958	A/Gull/Chile/7023-3/2022_|H5N1|_2022-12-07
EPI_ISL_17011959	A/Pelican/Chile/6958-1/2022_|H5N1|_2022-12-06
EPI_ISL_17011963	A/Pelican/Chile/6924-1/2022_|H5N1|_2022-12-06
EPI_ISL_17011964	A/Gull/Chile/7023-2/2022_|H5N1|_2022-12-07
EPI_ISL_17012018	A/Pelican/Chile/7087-1/2022_|H5N1|_2022-12-07
EPI_ISL_17015362	A/chicken/Czech_Republic/2750-3/2023_|H5N1|_2023-02-16
EPI_ISL_17033246	A/Peregrine_falcon/Netherlands/5/2023_|H5N1|_2023-02-06
EPI_ISL_17051025	A/chicken/BC/AIVPHL-23/2022_|H5N1|_2022-05-02
EPI_ISL_17051082	A/duck/BC/AIVPHL-135/2022_|H5N1|_2022-05-20
EPI_ISL_17051408	A/northern_shoveler/BC/AIVPHL-239/2022_|H5N1|_2022-09-12
EPI_ISL_17072111	A/African_penguin/South_Africa/702626_AP821/2022_|H5N1|_2022-11-07
EPI_ISL_17165500	A/cygnus_olor/Romania/17142_23VIR1145-1/2022_|H5N1|_2022-11-15
EPI_ISL_17165509	A/turkey/Romania/10281_23VIR1145-10/2023_|H5N1|_2023-01-27
EPI_ISL_17185876	A/black_vulture/Georgia/W22-674/2022_|H5N1|_2022-08-24
EPI_ISL_17185878	A/black_vulture/Virginia/W22-667D/2022_|H5N1|_2022-08-22
EPI_ISL_17214732	A/greylag_goose/England/321390/2023_|H5N1|_2023-02-14
EPI_ISL_17267428	A/large-billed_crow/Hokkaido/B005/2022_|H5N1|_2022-12-21
EPI_ISL_17317373	A/large-billed_crow/Hokkaido/B00C/2022_|H5N1|_2022-12-19
EPI_ISL_17353838	A/chicken/Magdalena/ICA-3503/2022_|H5N1|_2022-11-18
EPI_ISL_17414584	A/avian/Nigeria/115_22VIR3286-61/2022_|H5N1|_2022-01-28
EPI_ISL_17414588	A/avian/Nigeria/658_22VIR3286-8/2021_|H5N1|_2021-10-28
EPI_ISL_17414591	A/avian/Nigeria/684_22VIR3286-12/2021_|H5N1|_2021-11-19
EPI_ISL_17414594	A/avian/Nigeria/737_22VIR3286-25/2021_|H5N1|_2021-12-24
EPI_ISL_17414595	A/avian/Nigeria/738_22VIR3286-26/2021_|H5N1|_2021-12-24
EPI_ISL_17414596	A/avian/Nigeria/739_22VIR3286-27/2021_|H5N1|_2021-12-24
EPI_ISL_17414597	A/avian/Nigeria/741_22VIR3286-29/2021_|H5N1|_2021-12-28
EPI_ISL_17414600	A/avian/Nigeria/VRD-21-019_21RS744-61/2021_|H5N1|_2021-01-22

EPI_ISL_17414601	A/avian/Nigeria/VRD-21-020_21RS744-62/2021_|H5N1|_2021-01-22
EPI_ISL_17414608	A/avian/Nigeria/VRD-21-140_21VIR7423-19/2021_|H5N1|_2021-03-12
EPI_ISL_17414609	A/avian/Nigeria/VRD-21-174_21VIR7423-22/2021_|H5N1|_2021-03-21
EPI_ISL_17414610	A/avian/Nigeria/VRD-21-181_21VIR7423-2/2021_|H5N1|_2021-03-23
EPI_ISL_17414611	A/chicken/Nigeria/001_22VIR3286-36/2022_|H5N1|_2022-01-04
EPI_ISL_17414613	A/chicken/Nigeria/011_22VIR3286-40/2022_|H5N1|_2022-01-07
EPI_ISL_17414621	A/chicken/Nigeria/037_22VIR3286-49/2022_|H5N1|_2022-01-13
EPI_ISL_17414623	A/chicken/Nigeria/042_22VIR3286-51/2022_|H5N1|_2022-01-15
EPI_ISL_17414624	A/chicken/Nigeria/048_22VIR3286-52/2022_|H5N1|_2022-01-17
EPI_ISL_17414628	A/chicken/Nigeria/078_22VIR3286-57/2022_|H5N1|_2022-01-22
EPI_ISL_17414629	A/chicken/Nigeria/082_22VIR3286-58/2022_|H5N1|_2022-01-24
EPI_ISL_17414635	A/chicken/Nigeria/146_22VIR3286-66/2022_|H5N1|_2022-02-01
EPI_ISL_17414636	A/chicken/Nigeria/149_22VIR3286-67/2022_|H5N1|_2022-02-02
EPI_ISL_17414637	A/chicken/Nigeria/157_22VIR3286-68/2022_|H5N1|_2022-02-03
EPI_ISL_17414638	A/chicken/Nigeria/164A_22VIR3286-69/2022_|H5N1|_2022-02-04
EPI_ISL_17414642	A/chicken/Nigeria/653_22VIR3286-6/2021_|H5N1|_2021-10-26
EPI_ISL_17414643	A/chicken/Nigeria/664_22VIR3286-9/2021_|H5N1|_2021-11-05
EPI_ISL_17414644	A/chicken/Nigeria/686-H5B04_22VIR3286-16/2021_|H5N1|_2021-12-20
EPI_ISL_17414647	A/chicken/Nigeria/709_22VIR3286-15/2021_|H5N1|_2021-12-06
EPI_ISL_17414653	A/chicken/Nigeria/746_22VIR3286-32/2021_|H5N1|_2021-12-29
EPI_ISL_17414655	A/chicken/Nigeria/VRD-21-212_21VIR7423-5/2021_|H5N1|_2021-04-03
EPI_ISL_17414657	A/chicken/Nigeria/VRD-21-338_21VIR7423-29/2021_|H5N1|_2021-06-30
EPI_ISL_17414662	A/goose/Nigeria/VRD-21-293_21VIR7423-26/2021_|H5N1|_2021-05-28
EPI_ISL_17414663	A/peacock/Nigeria/VRD-21-187_21VIR7423-4/2021_|H5N1|_2021-03-25
EPI_ISL_17414664	A/poultry/Nigeria/VRD-21-135_21VIR7423-18/2021_|H5N1|_2021-03-10
EPI_ISL_17414668	A/guinea_fowl/Nigeria/VRD-21-169_21VIR7423-21/2021_|H5N1|_2021-03-21
EPI_ISL_17414669	A/Falco_peregrinus/Beijing/1/_2022_|H5N1|_2022-10-09
EPI_ISL_17415170	A/avian/Nigeria/271PT_22VIR3286-71/2021_|H5N1|_2021-01-01
EPI_ISL_17424639	A/blue-winged_teal/Texas/UGAI22-3189/2022_|H5N1|_2022-10
EPI_ISL_17424649	A/blue-winged_teal/Texas/UGAI22-2961/2022_|H5N1|_2022-09

EPI_ISL_17468386	A/Chile/25945/2023_|H5N1|_2023-03-24
EPI_ISL_17470102	A/chicken/Czech_Republic/3446_orig/2023_|H5N1|_2023-03-02
EPI_ISL_17471324	A/red-tailed_hawk/Kansas/W22-1226/2022_|H5N1|_2022-12-05
EPI_ISL_17514130	A/chicken/Austria/23006928-026/2023_|H5N1|_2023-01-20
EPI_ISL_17514136	A/coscoroba_swan/Austria/23005657-003/23_|H5N1|_2023-01-18
EPI_ISL_17521022	A/domestic_duck/England/049343/2023_|H5N1|_2023-03-30
EPI_ISL_17521049	A/black-headed_gull/England/053469/2023_|H5N1|_2023-03-23
EPI_ISL_17529372	A/duck/Egypt/RA19853OP/2021_|H5N1|_2021-12-15
EPI_ISL_17583227	A/Stone_Marten/Netherlands/EMC1/2022_|H5N1|_2022-01-16
EPI_ISL_17606814	A/mandarin_duck/Korea/H519/2021_|H5N1|_2021-11-05
EPI_ISL_17606815	A/mandarin_duck/Korea/WA622/2021_|H5N1|_2021-11-09
EPI_ISL_17606816	A/mandarin_duck/Korea/H552/2021_|H5N1|_2021-11-15
EPI_ISL_17606817	A/mallard/Korea/WA44/2022_|H5N1|_2022-01-13
EPI_ISL_17606818	A/wild_duck/Korea/H152/2022_|H5N1|_2022-02-08
EPI_ISL_17606819	A/duck/Korea/H535-2/2021_|H5N1|_2021-11-10
EPI_ISL_17606821	A/duck/Korea/H550/2021_|H5N1|_2021-11-16
EPI_ISL_17606822	A/duck/Korea/H554/2021_|H5N1|_2021-11-17
EPI_ISL_17606823	A/chicken/Korea/H559/2021_|H5N1|_2021-11-21
EPI_ISL_17606824	A/duck/Korea/H560/2021_|H5N1|_2021-11-22
EPI_ISL_17606831	A/duck/Korea/H621/2021_|H5N1|_2021-12-15
EPI_ISL_17606833	A/quail/Korea/H624/2021_|H5N1|_2021-12-17
EPI_ISL_17606834	A/chicken/Korea/H625/2021_|H5N1|_2021-12-20
EPI_ISL_17606835	A/chicken/Korea/H638/2021_|H5N1|_2021-12-23
EPI_ISL_17606837	A/duck/Korea/H649/2021_|H5N1|_2021-12-29
EPI_ISL_17606839	A/duck/Korea/H38/2022_|H5N1|_2022-01-14
EPI_ISL_17606844	A/duck/Korea/H79/2022_|H5N1|_2022-01-25
EPI_ISL_17606849	A/Korean_native_chicken/Korea/H97/2022_|H5N1|_2022-02-03
EPI_ISL_17606850	A/chicken/Korea/H124/2022_|H5N1|_2022-02-04
EPI_ISL_17606851	A/Korean_native_chicken/Korea/H127/2022_|H5N1|_2022-02-06
EPI_ISL_17606854	A/chicken/Korea/H130/2022_|H5N1|_2022-02-07

EPI_ISL_17606855	A/duck/Korea/H138/2022_|H5N1|_2022-02-08
EPI_ISL_17606859	A/chicken/Korea/H154-1/2022_|H5N1|_2022-02-13
EPI_ISL_17606860	A/chicken/Korea/H155/2022_|H5N1|_2022-02-12
EPI_ISL_17606861	A/duck/Korea/H196/2022_|H5N1|_2022-02-18
EPI_ISL_17606862	A/duck/Korea/H200/2022_|H5N1|_2022-02-21
EPI_ISL_17606863	A/duck/Korea/H232/2022_|H5N1|_2022-03-02
EPI_ISL_17606865	A/duck/Korea/H543/2021_|H5N1|_2021-11-14
EPI_ISL_17638448	A/chicken/Hokkaido/HU-B301/2023_|H5N1|_2023-04-06
EPI_ISL_17638492	A/chicken/Niger/288-22_23VIR3551-5/2022_|H5N1|_2022-12-18
EPI_ISL_17638493	A/chicken/Niger/12-23_23VIR3551-8/2023_|H5N1|_2023-01-12
EPI_ISL_17638499	A/goose/Niger/41-23_23VIR3551-23/2023_|H5N1|_2023-01-28
EPI_ISL_17638500	A/chicken/Niger/43-23_23VIR3551-25/2023_|H5N1|_2023-01-28
EPI_ISL_17638502	A/chicken/Niger/51-23_23VIR3551-32/2023_|H5N1|_2023-02-02
EPI_ISL_17638503	A/goose/Niger/59-23_23VIR3551-35/2023_|H5N1|_2023-02-06
EPI_ISL_17638504	A/chicken/Niger/67-23_23VIR3551-36/2023_|H5N1|_2023-02-10
EPI_ISL_17638505	A/guinea_fowl/Niger/71-23_23VIR3551-38/2023_|H5N1|_2023-02-14
EPI_ISL_17657734	A/chicken/England/062635/2023_|H5N1|_2023-04-28
EPI_ISL_17660073	A/owl/Peru/23-005629-001-original/2022_|H5N1|_2022-11-11
EPI_ISL_17660074	A/brown_booby/Peru/23-005629-003-original/2022_|H5N1|_2022-11-11
EPI_ISL_17679737	A/chicken/Italy/23VIR2802-7/2023_|H5N1|_2023-03-27
EPI_ISL_17679790	A/teal/Italy/22VIR10396-3/2022_|H5N1|_2022-10-26
EPI_ISL_17679801	A/seagull/Spain/4878-10_23VIR1687-18/2022_|H5N1|_2022-12-25
EPI_ISL_17710031	A/Black-Headed_Gull/England/259288/2023_|H5N1|_2023-04-14
EPI_ISL_17710042	A/Common_Buzzard/England/125155/2023_|H5N1|_2023-04-17
EPI_ISL_17716063	A/black-headed_gull/Austria/23025916-001/2023_|H5N1|_2023-02-28
EPI_ISL_17716064	A/black-headed_gull/Austria/23030829-001/2023_|H5N1|_2023-03-08
EPI_ISL_17716067	A/black-headed_gull/Austria/23045023-001/2023_|H5N1|_2023-04-04
EPI_ISL_17716080	A/mute_swan/Austria/23001568-001/2023_|H5N1|_2023-01-05
EPI_ISL_17732340	A/peregrine_falcon/Czech_Republic/7735-2_chymus/2023_|H5N1|_2023-05-17
EPI_ISL_17766057	A/Ezo_red_fox/Hokkaido/2/2023_|H5N1|_2023-04-12

EPI_ISL_17777527	A/gull/Peru/LIM-INS-006/2023_|H5N1|_2023-03-16
EPI_ISL_17777528	A/peruvian_booby/Peru/CAL-INS-007/2023_|H5N1|_2023-03-17
EPI_ISL_17777530	A/guanay_cormorant/Peru/CAL-INS-009/2023_|H5N1|_2023-03-17
EPI_ISL_17777532	A/sea_lion/Peru/TAC-INS-011/2023_|H5N1|_2023-03-07
EPI_ISL_17805986	A/chicken/Peru/AIS0546/2022_|H5N1|_2022-12-18
EPI_ISL_17805990	A/chicken/Peru/AIS0543/2022_|H5N1|_2022-12-01
EPI_ISL_17805996	A/chicken/Peru/AIS0549/2022_|H5N1|_2022-12-22
EPI_ISL_17805999	A/lion/Peru/AIS0554/2023_|H5N1|_2023-02-08
EPI_ISL_17806001	A/chicken/Peru/AIS0551/2022_|H5N1|_2022-12-12
EPI_ISL_17806003	A/pelican/Peru/AISA0464/2022_|H5N1|_2022-11-22
EPI_ISL_17821065	A/Black-headed_Gull/Netherlands/80/2023_|H5N1|_2023-05-24
EPI_ISL_17830838	A/Chroicocephalus_ridibundus/Belgium/01145_0006/2023_|H5N1|_2023-02-07
EPI_ISL_17831040	A/Falco_peregrinus/Belgium/04695_0002/2023_|H5N1|_2023-05-07
EPI_ISL_17885849	A/black_skimmer/Maule/240379/2023_|H5N1|_2023-03-06
EPI_ISL_17885869	A/goose/Araucania/239189-1/2023_|H5N1|_2023-02-28
EPI_ISL_17885870	A/chicken/OHiggins/241252-6/2023_|H5N1|_2023-03-12
EPI_ISL_17885874	A/chicken/Nuble/241557-1/2023_|H5N1|_2023-03-10
EPI_ISL_17885877	A/chicken/Nuble/240155/2023_|H5N1|_2023-03-06
EPI_ISL_17885878	A/chicken/Nuble/239136/2023_|H5N1|_2023-02-27
EPI_ISL_17885912	A/pelican/OHiggins/233721-1/2023_|H5N1|_2023-01-24
EPI_ISL_17885918	A/pelican/Nuble/233947-2/2023_|H5N1|_2023-01-26
EPI_ISL_17885924	A/pelican/Atacama/230158-2/2022_|H5N1|_2022-12-30
EPI_ISL_17885942	A/pelican/Tarapaca/227436-2/2022_|H5N1|_2022-12-09
EPI_ISL_17885944	A/pelican/Antofagasta/228244-2/2022_|H5N1|_2022-12-16
EPI_ISL_17885946	A/chicken/Araucania/241892-2/2023_|H5N1|_2023-03-14
EPI_ISL_17885948	A/humboldt_penguin/Coquimbo/239590/2023_|H5N1|_2023-02-28
EPI_ISL_17885950	A/great_egret/Araucania/240518/2023_|H5N1|_2023-03-07
EPI_ISL_17885951	A/gray_gull/Tarapaca/232825-1/2023_|H5N1|_2023-01-18
EPI_ISL_17885955	A/elegant_tern/Arica_y_Parinacota/229476-1/2022_|H5N1|_2022-12-24
EPI_ISL_17885964	A/turkey/Nuble/240489-1/2023_|H5N1|_2023-03-07

EPI_ISL_17885965	A/chiloe_wigeon/OHiggins/240893-2/2023_|H5N1|_2023-03-09
EPI_ISL_17885967	A/blackish_oystercatcher/OHiggins/240628/2023_|H5N1|_2023-03-06
EPI_ISL_17885970	A/turkey_vulture/Antofagasta/228252-1/2022_|H5N1|_2022-12-17
EPI_ISL_17885972	A/kelp_gull/Maule/239349/2023_|H5N1|_2023-03-01
EPI_ISL_17885973	A/tern/Maule/238507/2023_|H5N1|_2023-02-23
EPI_ISL_17885976	A/sea_lion/Arica_y_Parinacota/240270-1/2023_|H5N1|_2023-03-01
EPI_ISL_17885978	A/sanderling/Arica_y_Parinacota/240265/2023_|H5N1|_2023-03-03
EPI_ISL_17885980	A/sanderling/Arica_y_Parinacota/230758-1/2022_|H5N1|_2022-12-30
EPI_ISL_17885982	A/pelican/Valparaiso/234040/2023_|H5N1|_2023-01-25
EPI_ISL_17885983	A/pelican/Valparaiso/233450-1/2023_|H5N1|_2023-01-23
EPI_ISL_17949961	A/large-billed_crow/Kanagawa/1403C006/2023_|H5N1|_2023-03-10
EPI_ISL_17949986	A/large-billed_crow/Niigata/1502B004/2023_|H5N1|_2023-02-07
EPI_ISL_17950253	A/large-billed_crow/Hokkaido/0103E089/2023_|H5N1|_2023-03-30
EPI_ISL_17964940	A/sanderling/Oregon/23-011962-001-original/2023_|H5N1|_2023-04-13
EPI_ISL_17973458	A/wildbird-Fregata-magnificens/Ecuador/IC06-4590/2023_|H5N1|_2023-01-11
EPI_ISL_17985398	A/Black-headed_Gull/Netherlands/87/2023_|H5N1|_2023-06-16
EPI_ISL_17985406	A/Black-headed_Gull/Netherlands/96/2023_|H5N1|_2023-06-20
EPI_ISL_17996130	A/sandwich_tern/Italy/23VIR5828-2/2023_|H5N1|_2023-06-16
EPI_ISL_17999470	A/peregrine_falcon/Niigata/1510C001T/2022_|H5N1|_2022-10-16
EPI_ISL_18007232	A/large-billed_crow/Niigata/1503B017/2023_|H5N1|_2023-03-24
EPI_ISL_18032447	A/domestic_goose/Poland/H440/2022_|H5N1|_2022-12-17
EPI_ISL_18033169	A/domestic_duck/Poland/H70-T1/2023_|H5N1|_2023-01-16
EPI_ISL_18033182	A/domestic_duck/Poland/H101-T2/2023_|H5N1|_2023-02-03
EPI_ISL_18033206	A/black_headed_gull/Poland/MB142-T/2023_|H5N1|_2023-04-20
EPI_ISL_18033230	A/mute_swan/Poland/MB203-NJ/2023_|H5N1|_2023-05-11
EPI_ISL_18033235	A/common_tern/Poland/MB247-5/2023_|H5N1|_2023-05-30
EPI_ISL_18054502	A/Sea_Lion/Peru/LIM-SER036/2023_|H5N1|_2023-01-23
EPI_ISL_18054509	A/sea_lion/Peru/AQP-SER00K/2023_|H5N1|_2023-02-07
EPI_ISL_18065343	A/partridge/England/097407/2023_|H5N1|_2023-07-23
EPI_ISL_18066450	A/large-billed_crow/Miyazaki/4501A501/2023_|H5N1|_2023-01-06

EPI_ISL_18066451	A/whooper_swan/Tochigi/0902C010/2023_|H5N1|_2023-02-09
EPI_ISL_18066454	A/black-throated_crane/Tokyo/1302005b/2023_|H5N1|_2023-02-23
EPI_ISL_18066455	A/northern_goshawk/Shizuoka/2201D004/2023_|H5N1|_2023-01-07
EPI_ISL_18066456	A/Eurasian_Jay/Tochigi/0902A011/2023_|H5N1|_2023-02-20
EPI_ISL_18066457	A/large-billed_crow/Fukuoka/4002A005/2023_|H5N1|_2023-02-06
EPI_ISL_18071580	A/goose/Magadan/2272-5/2022_|H5N1|_2022-10-09
EPI_ISL_18075716	A/sandwich_tern/Spain/2740-23-2023_23VIR6502-23/2023_|H5N1|_2023-06-07
EPI_ISL_18075717	A/gull-billed_tern/Spain/2740-28-2023_23VIR6502-24/2023_|H5N1|_2023-06-07
EPI_ISL_18102701	A/feline/South_Korea/SNU-02/2023_|H5N1|_2023-07-04
EPI_ISL_18122446	A/black-headed-gull/Finland/7826/2023_|H5N1|_2023-07-11
EPI_ISL_18123937	A/black-headed-gull/Finland/7898/2023_|H5N1|_2023-07-13
EPI_ISL_18127713	A/wild_bird/Gambia/23_198_TJ008_23VIR5825-8/2023_|H5N1|_2023-04-01
EPI_ISL_18130247	A/large-billed_crow/Saitama/1101020/2023_|H5N1|_2023-01-05
EPI_ISL_18132189	A/Snow_Goose/Missouri/USDA-007698-003/2022_|H5N1|_2022-03-04
EPI_ISL_18132213	A/Tundra_Swan/Michigan/USDA-008506-009/2022_|H5N1|_2022-03-17
EPI_ISL_18132254	A/turkey/Indiana/USDA-003707-003/2022_|H5N1|_2022-02-07
EPI_ISL_18132257	A/Snow_Goose/South_Dakota/USDA-009478-006/2022_|H5N1|_2022-03-18
EPI_ISL_18132382	A/Mute_Swan/Michigan/USDA-011517-011/2022_|H5N1|_2022-04-11
EPI_ISL_18132441	A/chicken/Delaware/USDA-005260-001/2022_|H5N1|_2022-02-21
EPI_ISL_18132450	A/Ross's_Goose/Colorado/USDA-008935-005/2022_|H5N1|_2022-03-18
EPI_ISL_18132467	A/Snow_Goose/Iowa/USDA-008719-002/2022_|H5N1|_2022-03-22
EPI_ISL_18132490	A/Ross's_Goose/North_Dakota/USDA-011033-001/2022_|H5N1|_2022-04-05
EPI_ISL_18132491	A/turkey/Wisconsin/USDA-012973-001/2022_|H5N1|_2022-04-25
EPI_ISL_18132543	A/Wood_Duck/Minnesota/USDA-009381-001/2022_|H5N1|_2022-03-24
EPI_ISL_18132669	A/turkey/Minnesota/USDA-011141-003/2022_|H5N1|_2022-04-11
EPI_ISL_18132727	A/turkey/Minnesota/USDA-010995-002/2022_|H5N1|_2022-04-09
EPI_ISL_18132820	A/turkey/North_Carolina/USDA-009981-003/2022_|H5N1|_2022-03-31
EPI_ISL_18132823	A/turkey/South_Dakota/USDA-006792-001/2022_|H5N1|_2022-03-04
EPI_ISL_18132846	A/Bald_Eagle/Ohio/USDA-007697-001/2022_|H5N1|_2022-03-07
EPI_ISL_18132885	A/Bald_Eagle/North_Carolina/USDA-006530-002/2022_|H5N1|_2022-02-01

EPI_ISL_18132927	A/Bald_Eagle/Florida/USDA-007915-009/2022_|H5N1|_2022-02-23
EPI_ISL_18133046	A/Backyard_bird/Wyoming/USDA-009326-002/2022_|H5N1|_2022-03-29
EPI_ISL_18133123	A/Backyard_bird/New_York/USDA-010321-002/2022_|H5N1|_2022-04-04
EPI_ISL_18133135	A/Backyard_bird/Wisconsin/USDA-013061-005/2022_|H5N1|_2022-04-28
EPI_ISL_18133164	A/American_White_Pelican/Iowa/USDA-011101-003/2022_|H5N1|_2022-04-12
EPI_ISL_18133169	A/Lesser_Snow_Goose_White/Iowa/USDA-007679-009/2022_|H5N1|_2022-03-14
EPI_ISL_18133179	A/Backyard_bird/Minnesota/USDA-013221-001/2022_|H5N1|_2022-04-28
EPI_ISL_18133192	A/Mallard/North_Carolina/USDA-004008-006/2022_|H5N1|_2022-02-01
EPI_ISL_18133215	A/Backyard_bird/Michigan/USDA-011121-002/2022_|H5N1|_2022-04-11
EPI_ISL_18133226	A/Mallard/New_Hampshire/USDA-003417-053/2022_|H5N1|_2022-01-26
EPI_ISL_18133353	A/Duck/Indiana/USDA-011343-001/2022_|H5N1|_2022-04-12
EPI_ISL_18133359	A/Common_Goldeneye/Minnesota/USDA-010028-001/2022_|H5N1|_2022-03-31
EPI_ISL_18133422	A/goose/Florida/USDA-010682-001/2022_|H5N1|_2022-04-05
EPI_ISL_18133453	A/Gadwall/North_Carolina/USDA-001330-032/2022_|H5N1|_2022-01-08
EPI_ISL_18133495	A/Great_Horned_Owl/Wyoming/USDA-010158-002/2022_|H5N1|_2022-04-04
EPI_ISL_18226743	A/red_fox/Northern_Ireland/22185/2023_|H5N1|_2023-07-26
EPI_ISL_18241795	A/duck/Vietnam/HU16-DD125/2023_|H5N1|_2023-06-05
EPI_ISL_18241796	A/chicken/Vietnam/HU16-DD139/2023_|H5N1|_2023-09-05
EPI_ISL_18241797	A/muscovy_duck/Vietnam/HU16-DD144/2023_|H5N1|_2023-06-05
EPI_ISL_18245789	A/mute_swan/Poland/MB042-22_22VIR5675-6/2022_|H5N1|_2022-01-24
EPI_ISL_18265422	A/south_american_sea_lion/Peru/AQP-SER00R/2023_|H5N1|_2023-03-06
EPI_ISL_18265431	A/dolphin/Peru/PIU-SER002/2022_|H5N1|_2022-11-22
EPI_ISL_18265432	A/Sanderling/Peru/PIU-SER005/2022_|H5N1|_2022-11-22
EPI_ISL_18265433	A/pelican/Peru/PIU-SER013/2022_|H5N1|_2022-11-23
EPI_ISL_18265436	A/pelican/Peru/PIU-SER028/2022_|H5N1|_2022-11-24
EPI_ISL_18284461	A/chicken/Hokkaido/22A4T/2022_|H5N1|_2022-10-28
EPI_ISL_18284462	A/chicken/Hokkaido/22B3C/2022_|H5N1|_2022-11-07
EPI_ISL_18284467	A/duck/Wakayama/22A1T/2022_|H5N1|_2022-11-11
EPI_ISL_18284468	A/chicken/Aichi/22A3T/2022_|H5N1|_2022-12-05
EPI_ISL_18284469	A/chicken/Kagawa/22B2T/2022_|H5N1|_2022-11-22

EPI_ISL_18284472	A/duck/Chiba/22A3T/2022_|H5N1|_2022-11-26
EPI_ISL_18284475	A/chicken/Miyazaki/22B1T/2022_|H5N1|_2022-12-21
EPI_ISL_18292965	A/mink/Finland/709/2023_|H5N1|_2023-08-05
EPI_ISL_18304169	A/black-headed_gull/Leningrad_region/RII-WD299/2023_|H5N1|_2023-02-02
EPI_ISL_18310957	A/black-necked_swan/Uruguay/UDELAR-078-M2/2023_|H5N1|_2023-03-15
EPI_ISL_18310958	A/black-necked_swan/Uruguay/UDELAR-014-M3/2023_|H5N1|_2023-02-18
EPI_ISL_18310960	A/backyard_duck/Uruguay/UDELAR-124-M3/2023_|H5N1|_2023-04-16
EPI_ISL_18310962	A/backyard_chicken/Uruguay/UDELAR-127-M4/2023_|H5N1|_2023-04-17
EPI_ISL_18310965	A/backyard_chicken/Uruguay/UDELAR-047-M3/2023_|H5N1|_2023-03-06
EPI_ISL_18317757	A/european_herring_gull/Leningrad_region/RII-WD478S/2023_|H5N1|_2023-08-25
EPI_ISL_18321744	A/arctic-fox/Finland/HVVI_491_23_23VIR6803-1/2023_|H5N1|_2023-07-06
EPI_ISL_18325122	A/Curlew/Scotland/097010/2023_|H5N1|_2023-07-20
EPI_ISL_18371664	A/Belcher_gull/Peru/UNMSM-A267/2022_|H5N1|_2022-12
EPI_ISL_18371665	A/Guanay_cormorant/Peru/UNMSM-A275/2022_|H5N1|_2022-12
EPI_ISL_18371666	A/Peruvian_booby/Peru/UNMSM-A296/2022_|H5N1|_2022-12
EPI_ISL_18373217	A/Mandarin_duck/Korea/21WF119-1/2021_|H5N1|_2021-11-15
EPI_ISL_18373218	A/Mandarin_duck/Korea/21WF91-6/2021_|H5N1|_2021-11-16
EPI_ISL_18373219	A/Wild_duck/Korea/21WF124-4/2021_|H5N1|_2021-11-22
EPI_ISL_18373220	A/Baikal_teal/Korea/21WF192-9/2021_|H5N1|_2021-12-01
EPI_ISL_18373221	A/Wild_duck/Korea/21WF241-1/2021_|H5N1|_2021-12-05
EPI_ISL_18373222	A/Wild_duck/Korea/21WF213-1/2021_|H5N1|_2021-12-06
EPI_ISL_18373223	A/Wild_duck/Korea/21WF225-24/2021_|H5N1|_2021-12-07
EPI_ISL_18373227	A/Spot-billed_duck/Korea/21WS26-3/2022_|H5N1|_2022-01-18
EPI_ISL_18373228	A/Eurasian_wigeon/Korea/21WF549-3/2022_|H5N1|_2022-01-19
EPI_ISL_18373229	A/Wild_duck/Korea/21WF537-37/2022_|H5N1|_2022-01-21
EPI_ISL_18373231	A/Ruddy_shelduck/Korea/21WC127/2022_|H5N1|_2022-01-25
EPI_ISL_18373232	A/Spot-billed_duck/Korea/21WC138/2022_|H5N1|_2022-01-25
EPI_ISL_18373233	A/Wild_duck/Korea/21WF573-1/2022_|H5N1|_2022-01-26
EPI_ISL_18373234	A/Spot-billed_duck/Korea/21WC143/2022_|H5N1|_2022-01-27
EPI_ISL_18373238	A/Mallard/Korea/21WC183/2022_|H5N1|_2022-02-07

EPI_ISL_18373239	A/Gadwall/Korea/21WC196/2022_|H5N1|_2022-02-04
EPI_ISL_18373240	A/Bean_goose/Korea/21WC198/2022_|H5N1|_2022-02-10
EPI_ISL_18373245	A/White-fronted_goose/Korea/21WC244/2022_|H5N1|_2022-02-21
EPI_ISL_18373246	A/Great_egret/Korea/21WC249/2022_|H5N1|_2022-02-15
EPI_ISL_18373247	A/White-naped_crane/Korea/21WC257/2022_|H5N1|_2022-02-25
EPI_ISL_18373250	A/Bean_goose/Korea/21WC279/2022_|H5N1|_2022-03-04
EPI_ISL_18373252	A/Wild_bird_feces/Korea/21WF729-23/2022_|H5N1|_2022-03-03
EPI_ISL_18373254	A/White-fronted_goose/Korea/21WC305/2022_|H5N1|_2022-03-04
EPI_ISL_18373258	A/White-fronted_goose/Korea/21WC344/2022_|H5N1|_2022-03-16
EPI_ISL_18373261	A/White-fronted_goose/Korea/21WC361/2022_|H5N1|_2022-03-24
EPI_ISL_18378729	A/Chicken/Scotland/109635/2023_|H5N1|_2023-08-20
EPI_ISL_18497946	A/duck/Peru/LAM-INS-014/2023_|H5N1|_2023-09-26
EPI_ISL_18698459	A/Goose/Argentina/389-1/2023_|H5N1|_2023-02-11
EPI_ISL_18698460	A/Chicken/Argentina/464-4/2023_|H5N1|_2023-02-16
EPI_ISL_18698462	A/Chicken/Argentina/477-5/2023_|H5N1|_2023-02-18
EPI_ISL_18698464	A/Chicken/Argentina/481-2/2023_|H5N1|_2023-02-18
EPI_ISL_18698466	A/Chicken/Argentina/485-6/2023_|H5N1|_2023-02-19
EPI_ISL_18698469	A/Chicken/Argentina/491-2/2023_|H5N1|_2023-02-20
EPI_ISL_18698471	A/Chicken/Argentina/501-1/2023_|H5N1|_2023-02-20
EPI_ISL_18698474	A/Chicken/Argentina/506-2/2023_|H5N1|_2023-02-20
EPI_ISL_18698476	A/Chicken/Argentina/509-2/2023_|H5N1|_2023-02-21
EPI_ISL_18698478	A/Chicken/Argentina/556-6/2023_|H5N1|_2023-02-23
EPI_ISL_18698480	A/Chicken/Argentina/559-8/2023_|H5N1|_2023-02-22
EPI_ISL_18698482	A/Chicken/Argentina/578-2/2023_|H5N1|_2023-02-23
EPI_ISL_18698485	A/Avian/Argentina/579-5/2023_|H5N1|_2023-02-24
EPI_ISL_18698487	A/Avian/Argentina/586-4/2023_|H5N1|_2023-02-24
EPI_ISL_18698490	A/Chicken/Argentina/588-4/2023_|H5N1|_2023-02-24
EPI_ISL_18698492	A/Chicken/Argentina/606-1/2023_|H5N1|_2023-02-27
EPI_ISL_18698494	A/Chicken/Argentina/736-1/2023_|H5N1|_2023-03-03
EPI_ISL_18698497	A/Turkey/Argentina/753-1/2023_|H5N1|_2023-03-04

EPI_ISL_18698502	A/Chicken/Argentina/919-3/2023_|H5N1|_2023-03-13
EPI_ISL_18698504	A/Chicken/Argentina/1035-1/2023_|H5N1|_2023-03-16
EPI_ISL_18698505	A/Chicken/Argentina/1147-2/2023_|H5N1|_2023-03-21
EPI_ISL_18698506	A/Chicken/Argentina/1200-1/2023_|H5N1|_2023-03-27
EPI_ISL_18698507	A/Chicken/Argentina/1340-2/2023_|H5N1|_2023-03-31
EPI_ISL_18698508	A/Turkey/Argentina/1348-3/2023_|H5N1|_2023-03-31
EPI_ISL_18698510	A/Chicken/Argentina/1416-3/2023_|H5N1|_2023-04-03
EPI_ISL_18698511	A/Chicken/Argentina/1530-3/2023_|H5N1|_2023-04-12
EPI_ISL_18698512	A/Chicken/Argentina/1708-1/2023_|H5N1|_2023-04-19
EPI_ISL_18698513	A/Turkey/Argentina/1710-1/2023_|H5N1|_2023-04-17
EPI_ISL_18698514	A/Turkey/Argentina/1711-2/2023_|H5N1|_2023-04-19
EPI_ISL_18698515	A/Duck/Argentina/1712-5/2023_|H5N1|_2023-04-19
EPI_ISL_18698516	A/Avian/Argentina/1762-2/2023_|H5N1|_2023-04-21
EPI_ISL_18698517	A/Avian/Argentina/1790-5/2023_|H5N1|_2023-04-24
EPI_ISL_18698518	A/Chicken/Argentina/1976-2/2023_|H5N1|_2023-05-01
EPI_ISL_18698519	A/Chicken/Argentina/1984-4/2023_|H5N1|_2023-05-04
EPI_ISL_18698520	A/Chicken/Argentina/2034-5/2023_|H5N1|_2023-05-05
EPI_ISL_18698521	A/Chicken/Argentina/2049-3/2023_|H5N1|_2023-05-08
EPI_ISL_18698524	A/Chicken/Argentina/2305-1/2023_|H5N1|_2023-05-18
EPI_ISL_18698525	A/Chicken/Argentina/2305-6/2023_|H5N1|_2023-05-18
EPI_ISL_18698526	A/Chicken/Argentina/2483-3/2023_|H5N1|_2023-05-29
EPI_ISL_18698527	A/Chicken/Argentina/2796-2/2023_|H5N1|_2023-06-13
EPI_ISL_18698528	A/Chicken/Argentina/3346-1/2023_|H5N1|_2023-07-06
EPI_ISL_18698529	A/Chicken/Argentina/3657-2/2023_|H5N1|_2023-07-26
EPI_ISL_18698530	A/Chicken/Argentina/3695-3/2023_|H5N1|_2023-07-27
EPI_ISL_18698730	A/Chicken/Argentina/747-1/2023_|H5N1|_2023-03-04
EPI_ISL_18698731	A/Chicken/Argentina/895-1/2023_|H5N1|_2023-03-09
EPI_ISL_18698732	A/Chicken/Argentina/2016-2/2023_|H5N1|_2023-05-03
EPI_ISL_18698754	A/Sea_Lion/Argentina/3849-4/2023_|H5N1|_2023-08-08
EPI_ISL_18698755	A/Sea_Lion/Argentina/3893-1/2023_|H5N1|_2023-08-11

EPI_ISL_18755251	A/frigatebird/Rio_de_Janeiro/MAPA-1532N/2023_|H5N1|_2023-06-03
EPI_ISL_18755338	A/tern/Espirito_Santo/MAPA-1339N2/2023_|H5N1|_2023-05-15
EPI_ISL_18945315	A/South_American_tern/Argentina/RN-PB015/2023_|H5N1|_2023-08-26
EPI_ISL_18945316	A/South_American_sea_lion/Argentina/RN-PB013/2023_|H5N1|_2023-08-26
EPI_ISL_18945317	A/South_American_sea_lion/Argentina/RN-PB011/2023_|H5N1|_2023-08-26
EPI_ISL_18945319	A/South_American_sea_lion/Argentina/RN-PB007/2023_|H5N1|_2023-08-26
EPI_ISL_18945320	A/South_American_fur_seal/Argentina/RN-PB019/2023_|H5N1|_2023-08-26
EPI_ISL_2227278	A/goose/Netherlands/21028502-002/21028502/2021_|H5N1|_2021-05-09
EPI_ISL_4061484	A/chicken/Nigeria/VRD21-102_21VIR2370-424/2021_|H5N1|_2021-03-01
EPI_ISL_4061485	A/chicken/Nigeria/VRD21-109_21VIR2370-425/2021_|H5N1|_2021-03-01
EPI_ISL_4061491	A/chicken/Nigeria/VRD21-98_21VIR2288-6/2021_|H5N1|_2021-02-12
EPI_ISL_5463801	A/chicken/Saratov/29-07V/2021_|H5N1|_2021-09-30
EPI_ISL_5463804	A/chicken/Tyumen/33-45V/2021_|H5N1|_2021-10-07
EPI_ISL_5804789	A/great_skua/Scotland/B07779/2021_|H5N1|_2021-07-29
EPI_ISL_603135	A/Eurasian_Wigeon/Netherlands/5/2020_|H5N1|_2020-10-16
EPI_ISL_6328001	A/greylag_goose/Netherlands/21037497-001/2021_|H5N1|_2021-10-19
EPI_ISL_6328409	A/mute_swan/Czech_Republic/21312/2021_|H5N1|_2021-11-01
EPI_ISL_6761022	A/greylag_goose/Netherlands/21038567-002/2021_|H5N1|_2021-11-09
EPI_ISL_6829533	A/chicken/Kagoshima/21A6T/2021_|H5N1|_2021-11-12
EPI_ISL_6929958	A/turkey/Poland/H1910-T3/2021_|H5N1|_2021-11-01
EPI_ISL_6934175	A/domestic_duck/Poland/H1942-N/2021_|H5N1|_2021-11-07
EPI_ISL_6959592	A/mandarin_duck/Korea/WA585/2021_|H5N1|_2021-10-26
EPI_ISL_6959593	A/quail/Korea/H526/2021_|H5N1|_2021-11-08
EPI_ISL_711055	A/chicken/Netherlands/20019879-001005/2020_|H5N1|_2020-12-14
EPI_ISL_7626512	A/chicken/Czech_Republic/23404-2T/2021_|H5N1|_2021-11-25
EPI_ISL_7626532	A/chicken/Czech_Republic/23589-3/2021_|H5N1|_2021-11-29
EPI_ISL_7683138	A/red_fox/Netherlands/21040099-007/2021_|H5N1|_2021-12-03
EPI_ISL_7753193	A/Eurasian_wigeon/Germany-SH/AI05951/2021_|H5N1|_2021-10-14
EPI_ISL_7753251	A/barnacle_goose/Germany-SH/AI06005/2021_|H5N1|_2021-10-19
EPI_ISL_7753309	A/lesser_white-fronted_goose/Germany-MV/AI05973/2021_|H5N1|_2021-10-20

EPI_ISL_7753367	A/Eurasian_teal/Germany-BY/AI05977/2021_|H5N1|_2021-10-21
EPI_ISL_7778754	A/Withe-tiled_eagle/Estonia/TA2124126-1_21VIR10433-11/2021_|H5N1|_2021-10-12
EPI_ISL_7778768	A/Eurasian_eagle-owl/Finland/10617_21VIR7689-15/2021_|H5N1|_2021-08-01
EPI_ISL_8215689	A/Bar-headed_Goose/Tibet/XZ1131/2021_|H5N1|_2021-05-20
EPI_ISL_8515479	A/goose/Czech_Republic/22608-2/2021_|H5N1|_2021-11-18
EPI_ISL_8882172	A/turkey/Italy/21VIR10851/2021_|H5N1|_2021-12-06
EPI_ISL_9012457	A/chicken/England/053052/2021_|H5N1|_2021-10-24
EPI_ISL_9377021	A/goose/France/21P014207/2021_|H5N1|_2021-12-23
EPI_ISL_9603922	A/mute_swan/Czech_Republic/22684/2021_|H5N1|_2021-11-15
