# Supplementary material for: Detection and spread of high pathogenicity avian influenza virus H5N1 in the Antarctic Region
Source: Nat Commun. 2024 Sep 3;15:7433. doi: 10.1038/s41467-024-51490-8 (PMC11372179; doi:10.1038/s41467-024-51490-8)
Supplement: Supplementary file 3 — Reporting Summary [file 41467_2024_51490_MOESM3_ESM.pdf]

nature portfolio

Corresponding author(s): Ashley Banyard
Joe James

Last updated by author(s): 18/07/24

Reporting Summary

Nature Portfolio wishes to improve the reproducibility of the work that we publish. This form provides structure for consistency and transparency in reporting. For further information on Nature Portfolio policies, see our Editorial Policies and the Editorial Policy Checklist.

Statistics

For all statistical analyses, confirm that the following items are present in the figure legend, table legend, main text, or Methods section.

n/a	Confirmed	
☒	☐	The exact sample size (*n*) for each experimental group/condition, given as a discrete number and unit of measurement
☒	☐	A statement on whether measurements were taken from distinct samples or whether the same sample was measured repeatedly
☒	☐	The statistical test(s) used AND whether they are one- or two-sided<br>*Only common tests should be described solely by name; describe more complex techniques in the Methods section.*
☒	☐	A description of all covariates tested
☒	☐	A description of any assumptions or corrections, such as tests of normality and adjustment for multiple comparisons
☒	☐	A full description of the statistical parameters including central tendency (e.g. means) or other basic estimates (e.g. regression coefficient) AND variation (e.g. standard deviation) or associated estimates of uncertainty (e.g. confidence intervals)
☒	☐	For null hypothesis testing, the test statistic (e.g. *F*, *t*, *r*) with confidence intervals, effect sizes, degrees of freedom and *P* value noted<br>*Give P values as exact values whenever suitable.*
☐	☒	For Bayesian analysis, information on the choice of priors and Markov chain Monte Carlo settings
☒	☐	For hierarchical and complex designs, identification of the appropriate level for tests and full reporting of outcomes
☒	☐	Estimates of effect sizes (e.g. Cohen's *d*, Pearson's *r*), indicating how they were calculated

Our web collection on statistics for biologists contains articles on many of the points above.

Software and code

Policy information about availability of computer code

Data collection

No code was used for data collection, please see below for phylogenetic analysis

Data analysis

Assembly of the influenza A viral genomes was performed using a custom in-house pipeline as described previously but adapted for nanopore sequence reads. All influenza sequences generated and used in this study are available through the GISAID EpiFlu Database (https://www.gisaid.org). The remaining dataset was separated by segment and aligned using Mafft v7.52079, and manually trimmed to the open-reading frame using Aliview version 1.2680 The trimmed alignments were then used to a infer maximum-likelihood phylogenetic tree using IQ-Tree version 2.2.381 along with ModelFinder8 and 1,000 ultrafast bootstraps. For the time-resolved phylogenetic analysis, all HA sequences available from South America, and representatives from North America were combined with the sequences from South Georgia and the Falkland Islands and used to infer phylogeny using BEAST version 1.10.483 with the BEAGLE library. The Shapiro-Rambaut-Drummond-2006 (SRD06) nucleotide substitution model was implemented with a four-category gamma distribution model of site-specific rate variation and separate partitions for with the BEAGLE library. The Shapiro-Rambaut-Drummond-2006 (SRD06) nucleotide substitution model was implemented with a four-category gamma distribution model of site-specific rate variation and separate partitions for codon positions 1 plus 2 versus position 3 with the Hasegawa-Kishino-Yano (HKY) HKY substitution models on each with a strict clock and a coalescent GMRF Bayesian skyride tree prior. Three independent Markov Chain Monte Carlo (MCMC) runs were performed and combined using the Log Combiner tool in the BEAST package. Each chain consisted of 200,000,000 steps and was sampled every 20,000 steps, and the first 10% of samples were discarded as the burn-in. Discrete geographical transition events were reconstructed using a symmetric continuous-time Markov Chain model with an incorporated Bayesian stochastic search variable selection (BSSVS) to determine which transition rates sufficiently summarize connectivity86. SpreaD3 was used to determine the rates of transmission using a Bayes factor (BF) test. The BF represents the ratio of two competing statistical models, represented by their marginal likelihood, and, in this case, was used to determine the likelihood of transmission between geographical locations. The support of the BF for transmission was interpreted as described previously). BF and representative transitions related to South Georgia and the Falkland Islands were visualised on maps in R (v4.3.2) using the

packages GGPlot2, rnaturalearth, rnaturalearth data and sf. Nucleotide identity between sequences was determined as described previously82. Sequences were genotyped according to the USDA schema, using the GenoFLU tool (https://github.com/USDA-VS/GenoFLU)

For manuscripts utilizing custom algorithms or software that are central to the research but not yet described in published literature, software must be made available to editors and reviewers. We strongly encourage code deposition in a community repository (e.g. GitHub). See the Nature Portfolio guidelines for submitting code & software for further information.

Data

Policy information about availability of data

All manuscripts must include a data availability statement. This statement should provide the following information, where applicable:

- Accession codes, unique identifiers, or web links for publicly available datasets
- A description of any restrictions on data availability
- For clinical datasets or third party data, please ensure that the statement adheres to our policy

Sequence data is hosted on public accessible databases. All code used in data analysis is either published or hosted on github.

Research involving human participants, their data, or biological material

Policy information about studies with human participants or human data. See also policy information about sex, gender (identity/presentation), and sexual orientation and race, ethnicity and racism.

Reporting on sex and gender	N/A
Reporting on race, ethnicity, or other socially relevant groupings	N/A
Population characteristics	N/A
Recruitment	N/A
Ethics oversight	N/A

Note that full information on the approval of the study protocol must also be provided in the manuscript.

Field-specific reporting

Please select the one below that is the best fit for your research. If you are not sure, read the appropriate sections before making your selection.

☐ Life sciences ☐ Behavioural & social sciences ☒ Ecological, evolutionary & environmental sciences

For a reference copy of the document with all sections, see nature.com/documents/nr-reporting-summary-flat.pdf

Ecological, evolutionary & environmental sciences study design

All studies must disclose on these points even when the disclosure is negative.

Study description	Assessment of morality events on islands in the Antarctic region for evidence of high pathogenicity avian influenza infection. Critical assessment of potential incursion of a significant pathogen into a vulnerable ecosystem
Research sample	Birds and animals were sampled where mortalities or clinical disease was evident
Sampling strategy	Sampling was only conducted where safe to do so and to minimize potential pathogen spread by fomite transfer to healthy animals.
Data collection	Data was collected by staff on the ground to define location, species and approximate age.
Timing and spatial scale	Accessibility of these areas is challenging so samples were taken where mortality events, or clinical disease, was observed.
Data exclusions	No data were excluded
Reproducibility	All diagnostic assessments were undertaken within a UKAS accredited ISO:17025 framework and the National Reference Laboratory for Avian influenza in the UK
Randomization	This is not relevant as we were sampling at a disease outbreak in a sensitive site.
Blinding	This is not relevant as we were sampling at a disease outbreak in a sensitive site. However, samples submitted were blinded with respect to species and location for testing.

Did the study involve field work? ☒ Yes ☐ No

Field work, collection and transport

Field conditions	Weather as standard for location and season.
Location	Islands within the Antarctic region as described in the manuscript where all locations are stated.
Access & import/export	All samples collected with permissions from the Falkland Islands government and the government of South Georgia through collaboration with the British Antarctic survey.
Disturbance	Minimal disturbance as described above as critical to avoid further pathogens spread. Skilled ornithologists/field staff were collecting samples.

Reporting for specific materials, systems and methods

We require information from authors about some types of materials, experimental systems and methods used in many studies. Here, indicate whether each material, system or method listed is relevant to your study. If you are not sure if a list item applies to your research, read the appropriate section before selecting a response.

Materials & experimental systems

n/a	Involved in the study	
☒	☐	Antibodies
☒	☐	Eukaryotic cell lines
☒	☐	Palaeontology and archaeology
☐	☒	Animals and other organisms
☒	☐	Clinical data
☒	☐	Dual use research of concern
☒	☐	Plants

Methods

n/a	Involved in the study	
☒	☐	ChIP-seq
☒	☐	Flow cytometry
☒	☐	MRI-based neuroimaging

Animals and other research organisms

Policy information about studies involving animals; ARRIVE guidelines recommended for reporting animal research, and Sex and Gender in Research

Laboratory animals	N/A
Wild animals	Wild birds and mammal samples within the regions described as part of an outbreak assessment for infectious avian influenza virus
Reporting on sex	Carcasses of birds and animals that had died in te areas were sampled. Sex wasn't necessarily defined
Field-collected samples	Samples were collected from the field and shipped at ambient temperature to the Animal and Plant Health Agency lab in Weybridge, UK, for diagnostic assessment.
Ethics oversight	Only dead animals were sampled. However, all work undertaken on live animals in the UK of as part of a diagnostic evaluation abroad are done under strict ethical guidelines according to Home Office licenses

Note that full information on the approval of the study protocol must also be provided in the manuscript.

Plants

Seed stocks	N/A
Novel plant genotypes	N/A
Authentication	N/A
